# Endogenous SNAP-Tagging of Munc13‑1 for Monitoring Synapse Nanoarchitecture

**DOI:** 10.1021/jacsau.4c00946

**Published:** 2025-05-23

**Authors:** Maria Kowald, Sylvestre P. J. T. Bachollet, Fritz Benseler, Maria Steinecker, Moritz Boll, Sofia Kaushik, Tolga Soykan, Siqi Sun, Ramona Birke, Dragana Ilic, Nils Brose, Hanna Hörnberg, Martin Lehmann, Silvio O. Rizzoli, Johannes Broichhagen, Noa Lipstein

**Affiliations:** † 28417 Leibniz-Forschungsinstitut für Molekulare Pharmakologie (FMP), 13125 Berlin, Germany; ‡ 28282 Max Planck Institute for Multidisciplinary Sciences, 37075 Göttingen, Germany; § Max Delbrück Center for Molecular Medicine in the Helmholtz Association (MDC), 13125 Berlin, Germany; ∥ Department of Neuro- and Sensory Physiology, University Medical Center, 37073 Göttingen, Germany; ⊥ Excellence Cluster Multiscale Bioimaging (MBExC), 37073 Göttingen, Germany; # Center for Biostructural Imaging of Neurodegeneration (BIN), 27177 University Medical Center Göttingen, 37075 Göttingen, Germany

**Keywords:** Active zone, Munc13-1, Unc13a, Synapse, SNAP tag, SBG-SiR-d12

## Abstract

Synaptic function is governed by highly regulated protein machineries, whose abundance and spatial localization change continually. Studies to determine dynamic changes in synaptic protein nanoarchitecture typically rely on immunolabeling or on the expression of fluorescent proteins. The former employs chemical fluorophores and signal amplification but requires fixation. The latter enables monitoring of proteins by live microscopy but uses suboptimal fluorophores. Self-labeling tags have been introduced to combine the advantages of these two approaches, and here we introduce a knock-in mouse line where the essential presynaptic protein Munc13–1 is endogenously fused to the self-labeling SNAP tag. We demonstrate efficient Munc13–1-SNAP labeling in fixed cultured neurons and in brain sections by various SNAP dyes, as well as by a novel far-red and cell impermeable compound, SBG-SiR-d12. We introduce and characterize SBG-SiR-d12 as a highly efficient dye for SNAP-tag labeling of extracellular epitopes and of intracellular proteins such as Munc13–1 in fixed and permeabilized tissue. Finally, we show that Munc13–1-SNAP can be labeled in living neurons and monitored through live-cell imaging using confocal and super resolution microscopy. We conclude that the Unc13a^SNAP^ mouse line is a useful tool for labeling the presynaptic compartment and for the analysis of presynaptic nanoarchitectural dynamics, with potential for wide adoption.

## Graphical abstract

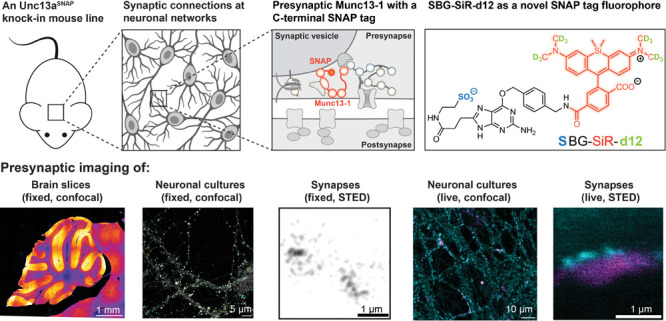

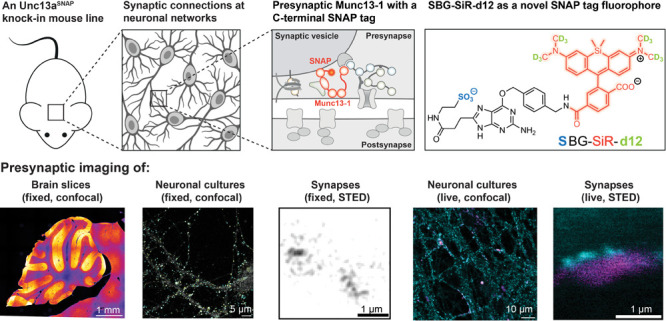

## Introduction

Dynamic changes in protein copy numbers, complex composition, and nanoscale organization often follow alterations in cellular activity levels. To reliably monitor proteins in time and space, efficient labeling strategies have been developed. A key development has been the introduction of fluorescent protein tags (e.g., green fluorescent protein, GFP^1,2^ ). However, and despite continuous improvements, the photophysical properties of fluorescent proteins often fall short compared to chemical dyes in terms of brightness and stability.^3,4^ To address this limitation, self-labeling tags were created: engineered protein tags that can be genetically encoded and covalently bind bioorthogonal synthetic probes. Belonging to this group, the SNAP tag^5^ is an engineered *O* ^6^-alkylguanine-DNA alkyltransferase that binds *O* ^6^-benzylguanine (BG) derivatives in a covalent, nonreversible manner.^6–8^ As such, proteins fused to a SNAP tag can be visualized in live cells or fixed tissue by the addition of a BG-fluorophore conjugate. This mode of protein labeling is advantageous as an alternative to genetic conjugation with fluorescent protein tags because it enables the flexible attachment of bright and stable dyes to the protein of interest, in the living cell, and at a time of choice. The size of the SNAP tag (∼19 kDa) brings the fluorophore in proximity to the protein of interest,^9,10^ which may be advantageous in super-resolution microscopy.^11^ Moreover, flexible use of chemical dyes enables pulse-chase labeling^12–15^ or signal multiplexing.^16^ The toolkit for SNAP tag labeling is rapidly expanding: BG derivatives carrying an array of fluorophores are available, and several chemical modifications of the BG-dye conjugates have been introduced to modify their properties, for example to make the conjugate membrane impermeable for extracellular labeling^13^ or to boost labeling kinetics.^17^

Here, we opted to leverage the advantages of self-labeling tags and develop novel tools to monitor synapses. Munc13–1 (protein unc-13 homologue A, encoded by the *Unc13a* gene) is a presynaptic protein with a central function in the preparation of synaptic vesicles (SVs) for fast exocytosis.^18,19^ Munc13–1 is expressed in the majority of neuronal subtypes in the central and peripheral nervous system, as well as in some neurosecretory cell types including chromaffin cells or insulin-releasing beta-pancreatic cells.^20,21^ In neurons, Munc13–1 resides in the active zone, a protein-dense compartment at the presynaptic membrane where SVs undergo fusion, and it is absolutely essential for synaptic transmission. At the molecular level, Munc13–1 catalyzes the formation of SNARE complexes, that link the SV membrane with the presynaptic plasma membrane, thus making SVs fusion-competent.^22,23^ Munc13–1 function sets multiple synapse properties, including synaptic strength, the release probability of SVs, the rate of SV replenishment after depletion, and synaptic plasticity.^19,23–32^ In humans, genetic variations in the *UNC13A* gene are associated with a neurodevelopmental syndrome,^33^ and noncoding intronic variants are among the strongest genetic risk factors for the neurodegenerative conditions amyotrophic lateral sclerosis (ALS) and frontotemporal dementia (FTD).^34,35^

Alongside changes in its function, the arrangement of Munc13–1 molecules at the active zone has been deemed critical for shaping synaptic transmission properties. In *D. melanogaster* neuromuscular junction synapses, the Munc13–1 ortholog Unc13a forms rings ∼70 nm around voltage-gated Ca^2+^ channels, contributing to the strong temporal coupling of synaptic transmission and Ca^2+^ influx triggered by an action potential.^36,37^ Upon synapse silencing, Unc13a expression is altered, with results pointing to an increase in the expression levels of Unc13a^38^ and/or compaction of the already-available Unc13a,^39^ both associated with a homeostatic increase in synaptic strength. In mammalian synapses, Munc13–1 is arranged in nanoclusters and the number of nanoclusters is positively correlated with the strength of glutamate release.^40^ Cryo-EM analysis and models based on the crystal structure of Munc13–1 promote the view that Munc13–1 forms hexameric rings surrounding one synaptic vesicle, acting in a cooperative manner to drive fusion.^41,42^ These rings have not been resolved in synapses yet, and, in general, tools are still lacking to visualize dynamic changes of the Munc13–1 nanoarchitecture in mammalian synapses.

Here, we present a novel CRISPR/Cas9 knock-in mouse line (Unc13a^SNAP^), in which we inserted a SNAP tag cassette at the endogenous *Unc13a* gene locus, to generate a Munc13–1 protein variant that is C-terminally fused to a SNAP tag (Munc13-1-SNAP). We validate this mouse line and interrogate neurons via confocal, stimulated emission-depletion (STED) microscopy, and live cell imaging at confocal and super-resolution. Due to the complex nature of cultured neurons and brain tissue used, and because Munc13–1 is a protein with a moderate to low expression level,^43^ we evaluated the performance of several SNAP tag substrates for efficient labeling. We tested the bright and stable far-red SNAP dyes BG-JF_646_ ^44^ and BG-SiR-d12,^45^ and, in addition, their membrane impermeable variants, SBG-JF_646_ ^13^ and SBG-SiR-d12, the synthesis and characterization of which we present here. Munc13–1 is a cytosolic protein and should not be labeled by membrane impermeable dyes, but given that some of our experiments were dealing with fixed and permeabilized preparations, we hypothesized that the charge originally installed to prevent membrane permeability may be useful in reducing background levels by repulsion. We report here that SBG-SiR-d12 successfully labels Munc13-1-SNAP in fixed cultured neurons and brain slices, with excellent performance in terms of brightness, specificity, and signal-to-noise ratio. Labeling using SBG-JF_646_, however, produced a substantial degree of background. We conclude that repurposing SBG-SiR-d12 for staining in permeabilized preparations is a promising approach to enhance labeling quality in complex samples, in cases where membrane permeability is irrelevant. We also conclude that the Unc13a^SNAP^ mouse line enables the detection of synapses and active zones at multiple scales in live and fixed neurons, and thus may be used to characterize their rapid dynamics and plasticity *in vivo* or *in vitro*.

## Results

### Generation and Validation of the Unc13a^SNAP^ Knock-In Mouse Line

We opted to generate a mouse model where endogenous Munc13–1 is C-terminally fused to a SNAP tag, with the two protein modules separated by a short and flexible 9 amino acid long linker (sequence: (GGS)_3_; Figure 1A). Supporting our design strategy, we relied on a previously generated knock-in mouse line where an enhanced yellow fluorescent protein (YFP) was added C-terminally to the Unc13a sequence.^46^ A structural model of Munc13–1-SNAP generated by AlphaFold3 ^47^ (Figure 1B) predicts three important features; i.e., (1) the addition of the SNAP tag likely does not change the Munc13–1 structure, (2) the SNAP tag likely does not exhibit protein–protein interactions with Munc13–1, and, importantly, (3) the C-terminal C2C domain likely remains structured and accessible to protein–protein or protein–lipid interactions, both of which are critical for Munc13–1 function in SV priming and thus for setting the strength of neurotransmission.^48–50^

**Figure 1:**
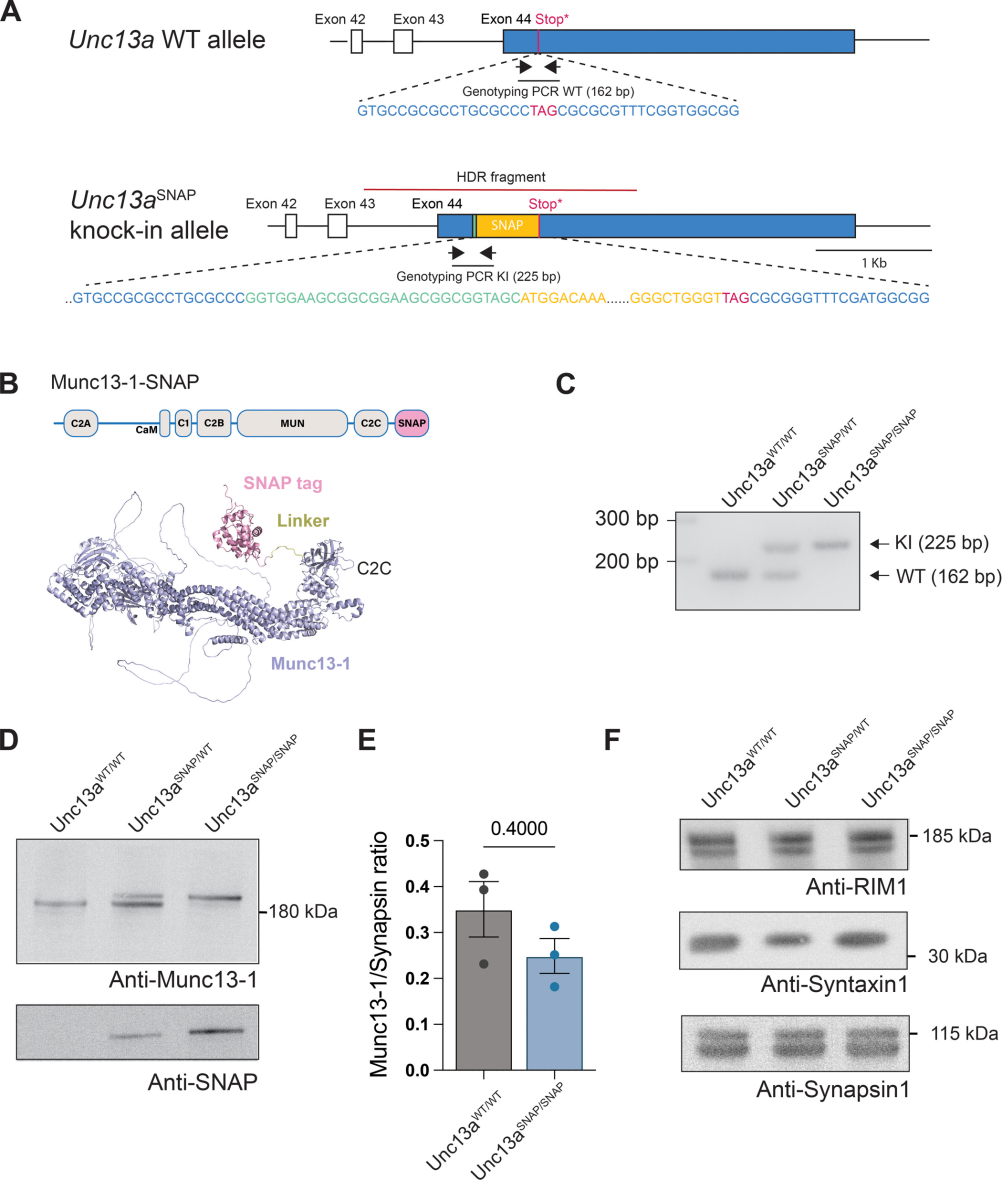
Validation of the Unc13a^SNAP^mouse line. (A) Scheme of the 3′ region of the *Unc13a* gene in WT and in targeted, Unc13a^SNAP^ mice, including the DNA sequences at the site of the SNAP tag insertion. Black arrows illustrate the location of the genotyping oligonucleotides, and a black line illustrates the PCR fragment. The linker, stop codon, and genotyping oligonucleotides are not drawn to scale. (B) Illustration of the Munc13–1 domain structure in the Unc13a^SNAP^ mice (top) and AlphaFold3 structural prediction of the Munc13–1-SNAP protein (down). (C) Genotyping PCR results for the indicated genotypes. (D) Western blot analysis of Munc13–1 (upper blot) and the SNAP tag (lower blot) in brain synaptosome homogenates from WT, heterozygous and homozygous Unc13a^SNAP^ mice. (E) Quantification of Munc13–1 levels from samples as in (D). Data represents mean ± SEM from three independent experiments, Mann–Whitney test for statistical significance. (F) Western blot analysis of RIM1, Syntaxin 1 and Synapsin 1 in brain homogenates from WT, heterozygous and homozygous Unc13a^SNAP^ mice.

The Unc13a^WT^ allele (Figure 1A) was targeted by site-directed CRISPR-Cas9 mutagenesis. The correct integration of the SNAP tag sequence was validated by long-range location PCRs (see Materials and Methods and Supporting Information for further information). In subsequent PCR analysis in genomic DNA from wild-type (WT), heterozygous (Unc13a^WT/SNAP^), and homozygous (Unc13a^SNAP/SNAP^) knock-in mice, we were able to amplify a DNA fragment spanning the last *Unc13a* exon and the SNAP tag cassette sequence (Unc13a^SNAP^ allele), confirming correct integration and enabling routine mouse genotyping (Figure 1C and Supporting Information).

The resulting homozygous Unc13a^SNAP^ mice (Unc13a^em1(SNAP)Bros^) were viable and fertile and exhibited no observable changes in survival, breeding performance, or cage behavior. Monitoring the well-being of the mice to adulthood (up to 8 months of age), we did not observe burden inflicted by the genetic modification. Because Munc13–1 loss results in perinatal lethality shortly after birth,^26^ we conclude that the Munc13–1-SNAP fusion protein is functional.

Next, we evaluated the expression levels of Munc13–1 by Western blot analysis in crude synaptosome fractions (P2) from WT, heterozygous, and homozygous mouse brains. We found a nonsignificant, mild reduction in the expression levels of Munc13–1-SNAP in comparison to the Munc13–1 WT protein (Figure 1D, E). Interestingly, in samples from heterozygous mice, the expression levels of the WT Munc13–1 protein appeared higher than that of the tagged Munc13–1 (Figure 1D, middle lane), which may indicate slightly reduced stability of the tagged protein in the presence of the WT form. This phenomenon has also been reported for the Munc13–1-eYFP fusion protein,^46^ and should be considered when working with heterozygous mice. Next, we established the expression of the SNAP tag cassette in samples from heterozygous and homozygous Unc13a^SNAP^ mice (Figure 1D), and excluded the presence of truncated Munc13–1-SNAP protein fragments, highlighting the stability of the tagged protein variant (Supporting Information, Figure S2). Finally, we found no change in the expression level of the major Munc13–1 interacting proteins Syntaxin 1A/B and RIM1 (regulating synaptic membrane exocytosis protein 1), as well as in the levels of Synapsin 1 in synaptosomal fractions from Unc13a^SNAP/SNAP^ mouse brains and WT littermate samples (Figure 1F). To confirm that the Munc13–1-SNAP fusion protein is fully functional, we conducted an electrophysiological analysis of synaptic transmission in glutamatergic excitatory neurons obtained from WT or Unc13a^SNAP/SNAP^ littermate brains. We did not observe statistically significant changes in the pattern or in the magnitude of synaptic transmission parameters, with the exception of a statistically significant increase in the frequency of miniature excitatory postsynaptic currents frequency (Supporting Information, Figure S1). We conclude that the genetic integration of a SNAP tag at the C-terminus of Munc13–1 does not lead to overt changes in Munc13–1 function or expression.

To provide additional evidence to support the proper expression of Munc13–1-SNAP in Unc13a^SNAP^ neurons, we performed immunocytochemical analysis in hippocampal neuron cultures from littermate WT and Unc13a^SNAP/SNAP^ mouse brains (Figure 2A, B; Table S1). We immunolabeled the neurons with (1) an antibody against the Munc13–1 protein, (2) an antibody against Bassoon, a presynaptic active zone marker, and (3) an antibody against MAP2, to label the dendritic extensions of the neuron. We quantified the expression levels of Munc13–1 in WT and in Unc13a^SNAP/SNAP^ samples based on the Munc13–1 signal intensity within regions of interest defined by the Bassoon signal, and found no changes between samples (Figure 2C). We also quantified the intensity of the signal arising from the labeling of Bassoon and found no differences between the two genotypes (Figure 2D), indicating that the expression of Munc13–1-SNAP does not change the expression levels of Bassoon. To evaluate whether Munc13–1-SNAP is correctly localized in the active zone, we determined the colocalization coefficient between the Bassoon and Munc13–1 signals (Figure 2E). We found a high degree of colocalization in both genotypes (mean Pearson’s correlation coefficient values, WT, 0.545 ± 0.014, n = 55 images; Unc13a^SNAP/SNAP^, 0.53 ± 0.017, n = 54 images; Mann–Whitney test, see Supporting Information Figure S5 for data plotted per regions of interest (ROIs)). We conclude that Munc13–1-SNAP is comparably expressed and localized properly in presynaptic active zones.

**Figure 2:**
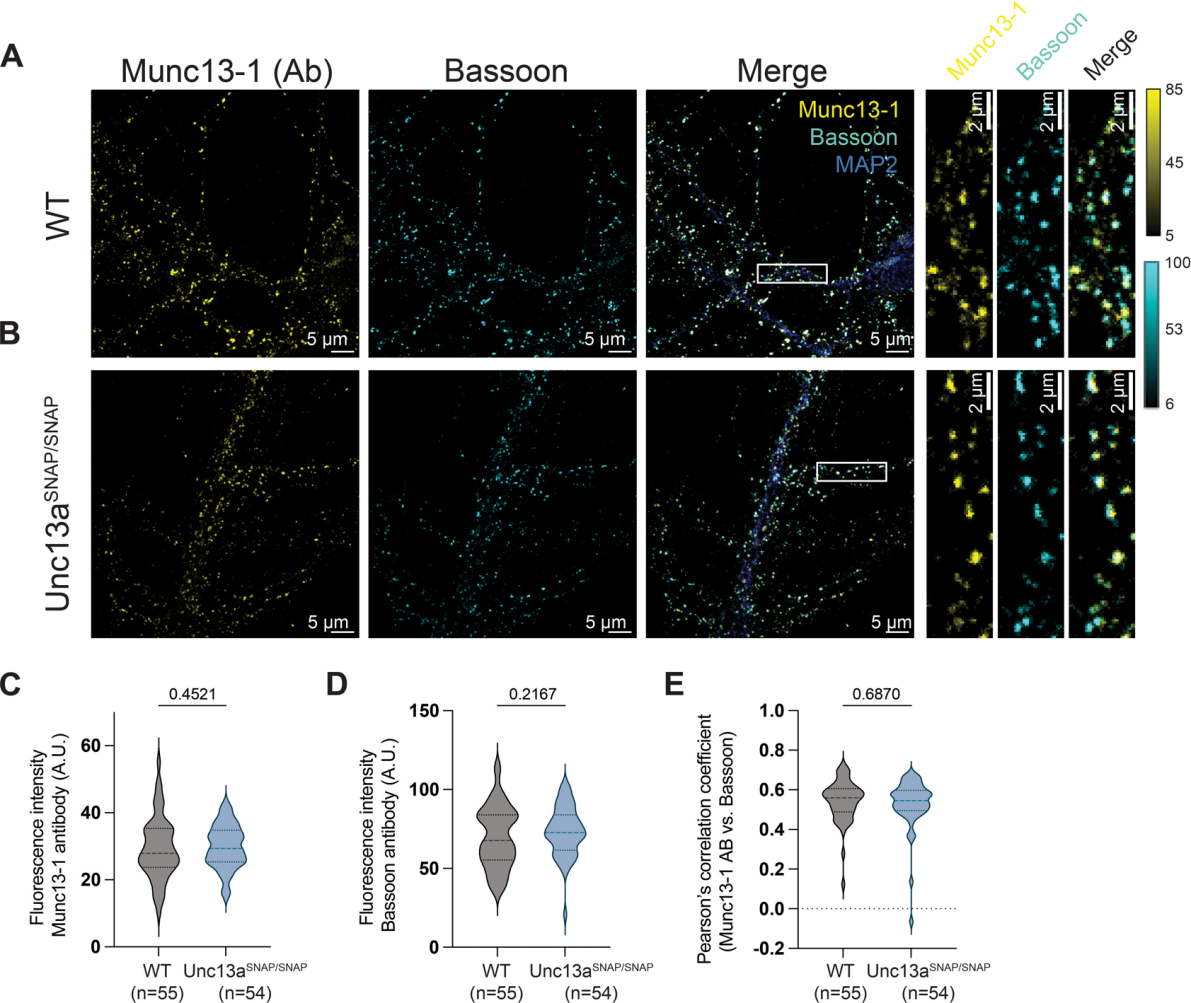
Validation of Munc13–1 localization in hippocampal neurons of Unc13a^SNAP^mice. Example images of cultured hippocampal neurons from WT (A) and Unc13a^SNAP/SNAP^ mouse brains (B), immunolabeled with antibodies (Ab) against Munc13–1, the active zone marker Bassoon, and MAP2 (scale bar: 5 μm; inset, 2 μm). On the right: magnification of the regions indicated by white boxes in the merged image. Quantification of (C) the fluorescence signal intensity arising from antibody labeling of Munc13–1 and (D) the fluorescence signal intensity arising from antibody labeling of Bassoon in neurons from WT and Unc13a^SNAP/SNAP^ mice, from images as in A and B. (E) Colocalization of Munc13–1 within Bassoon-labeled regions of interest, evaluated according to the Pearson’s correlation coefficient. In all violin plots, lines represent the median and 25% and 75% quartiles. Statistical significance was evaluated using a two-tailed Mann–Whitney test, n values represent the number of analyzed images, which were obtained from 3 independent experiments per condition (see Table S1 for a summary of the parameters used during microscopy experiments). A.U.: arbitrary units.

### The Development of SBG-SiR-d12

Following the knock-in mouse line validation , we next tested the efficiency of Munc13–1 labeling via the SNAP tag in fixed cultured primary neurons. In selecting the SNAP dyes, we opted for bright, stable, and far-red dyes (Figure 3A). Silicon Rhodamine (SiR), and the next generation fluorophores JF_646_ and SiR-d12, were developed to exhibit boosted brightness without loss of resolution.^44,45^ Conjugated to BG (i.e., BG-JF_646_ and BG-SiR-d12), these dyes can be used for SNAP tag labeling. To improve the specificity of staining, sulfonated BG (SBG) substrates have been created^13^ and benchmarked in complex tissue.^51^ The SBG moiety was originally designed for extracellular protein labeling, as it renders the conjugates membrane-impermeable. Thus, SBG-dye conjugates are not anticipated to label Munc13–1-SNAP, which is a cytosolic protein. Nonetheless, with the knowledge that the SBG moiety renders the conjugate less lipophilic, thus improving water solubility over prolonged periods of time,^10^ we considered that the charged SBG-dye conjugate might reduce background levels through surface repulsion and generate less nonspecific deposits. Cytosolic proteins could still be labeled if the sample had been fixed and permeabilized prior to the SNAP dye application. We report here the synthesis and validation of a novel membrane impermeable SNAP dye, SBG-SiR-d12 (see Materials and Methods and Supporting Information). We were able to obtain and validate SBG-SiR-d12 against a cohesive palette of far-red dyes for SNAP tag labeling (Figure 3A).

**Figure 3:**
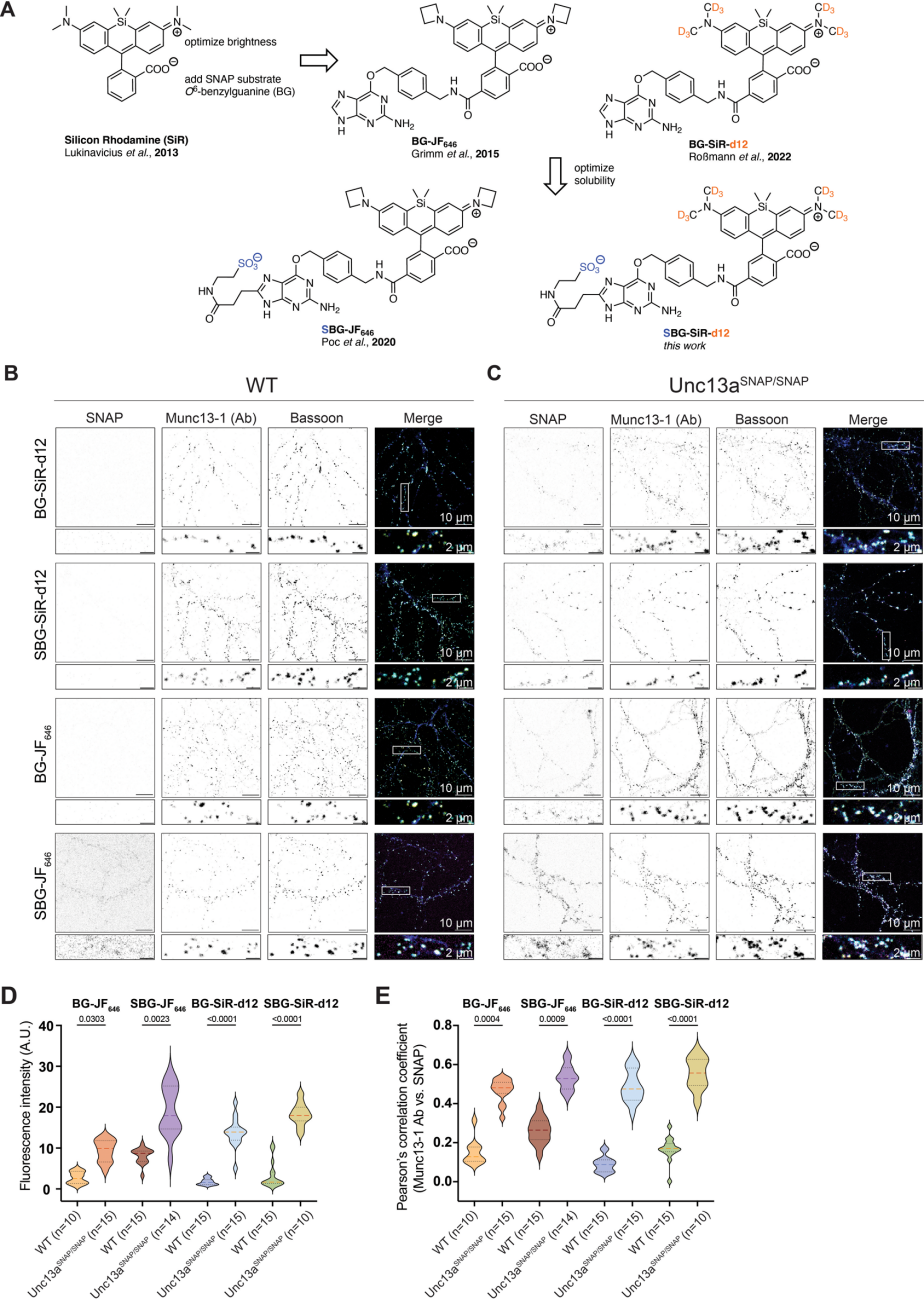
Development of SBG-SiR-d12 and validation of the SNAP tag functionality in cultured, fixed hippocampal Unc13a^SNAP^ neurons. (A) Chemical structures of silicone rhodamine variants for SNAP tag labeling, including SBG-SiR-d12 developed here. (B,C) Example images of fixed cultured hippocampal neurons from WT (B) and Unc13a^SNAP/SNAP^ (C) mouse brains, immunolabeled with the SNAP tag compounds BG-JF_646_, SBG-JF_646_, BG-SiR-d12, and SBG-SiR-d12, as well as with antibodies (Ab) against Munc13–1, Bassoon, and MAP2 (scale bar: 10 μm; inset, 2 μm). The location of the magnified regions below each image are indicated by the white box in the merged image. (D) Quantification of the fluorescence signal intensity arising from Munc13–1-SNAP labeling by BG-JF_646_, SBG-JF_646_, BG-SiR-d12, SBG-SiR-d12, in neurons from WT and Unc13a^SNAP/SNAP^ mice. (E) Colocalization of signals arising from simultaneous Munc13–1 labeling by an anti-Munc13–1 antibody and via the SNAP tag, using Pearson’s correlation coefficient. In the violin plots, lines represent the median and 25% and 75% quartiles. In all figures, n represents the number of images analyzed, and a two-sided Kruskal–Wallis test followed by Dunn’s test for multiple comparisons was used to determine statistical significance. Data was obtained in three independent experiments (Table S1). A.U.: arbitrary units.

To test for the functionality and membrane (im)­permeability of SBG-SiR-d12, HEK293 cells were transfected with a construct encoding for the fusion protein SNAP-TM-HTP, where an extracellular SNAP tag is separated by a transmembrane (TM) domain from an intracellular Halo Tag protein (HTP)^52^ (Supporting Information, Figure S3A). We then applied either BG-SiR-d12 (upper panel) or SBG-SiR-d12 (lower panel), together with chloroalkane-JF_519_ (CA-JF_519_), which is used to control for the expression of the SNAP-TM-HTP construct. Comparing BG-SiR-d12 and SBG-SiR-d12, we observed that the staining pattern of SBG-SiR-d12 is membrane-restricted, while BG-SiR-d12 also labels a cytosolic protein pool (e.g., membrane proteins still residing at the endoplasmic reticulum). This result suggests that SBG-SiR-d12 is less membrane permeable than BG-SiR-d12.

Next, we performed *in vitro* measurements to characterize the pH sensitivity and tentative background staining behavior of SBG-JF_646_ and SBG-SiR-d12. We subjected 100 nM SBG-SiR-d12 or SBG-JF_646_ to a pH titration (Supporting Information, Figure S3B) to gain information about their fluorogenicity. We found SBG-JF_646_ to be pH sensitive at more basic conditions while SBG-SiR-d12 was insensitive in a pH range from 4.2 to 9.2 (Supporting Information, Figure S3B). In a parallel experiment, we titrated BSA up to a concentration of 10 mg/mL, and found SBG-JF_646_ to give higher values in fluorescence polarization, indicating an increased tendency toward unspecific binding (Supporting Information, Figure S3C).

### Labeling of Munc13–1-SNAP in Fixed Samples Using BG-Dye Compounds

We used primary neuronal cultures from WT and Unc13a^SNAP/SNAP^ mice, and tested BG-JF_646,_ SBG-JF_646_, BG-SiR-d12 and SBG-SiR-d12 for their efficiency in labeling Munc13–1-SNAP. We immunolabeled fixed and permeabilized neurons with antibodies against Munc13–1, Bassoon and MAP2, and applied one of the BG-dye conjugates mentioned above (Figure 3B,C; Supporting Information, Table S1). Neurons from Unc13a^SNAP/SNAP^ mice were efficiently labeled by all four SNAP-targeted dyes (Figure 3C, panel “SNAP”). In WT, negative control neurons, we did not observe unspecific labeling, except in the case of SBG-JF_646_, where substantial background was observed (Figure 3B, left panel “SNAP”). The unspecific stickiness (Supporting Information, Figure S3C) might be the cause for the less crisp performance of SBG-JF_646_ compared to SBG-SiR-d12, potentially also in combination with the pH sensitivity. We analyzed the signal intensity arising from the SNAP tag labeling (Figure 3D), and found that the fluorescent signal was strongest for SBG-JF_646_ and SBG-SiR-d12 in knock-in synapses. Importantly, the best signal to background ratio, which we define here as the fold-change in the median value of the signal intensity between Unc13a^SNAP/SNAP^ synapses and WT synapses for each SNAP dye, was superior for SBG-SiR-d12 (BG-JF_646,_ 3.8-fold; SBG-JF_646_, 2.05-fold; BG-SiR-d12, 8.4-fold; SBG-SiR-d12, 10.6-fold).

To demonstrate labeling specificity, we assessed the colocalization between signals arising from labeling of the same antigen: the signal for Munc13–1 arising from the SNAP tag labeling and the signal of Munc13–1 arising from the antibody labeling. We found, as expected, a high degree of colocalization, which was similar for all dyes tested (Figure 3E). We conclude that the SNAP tag conjugated to Munc13–1 is functional and enables specific and bright labeling of Munc13–1 in fixed tissue at confocal resolution. SBG-SiR-d12 has emerged as the dye of choice to label the SNAP tag in permeabilized fixed samples.

We then tested whether the Unc13a^SNAP^ mice can be useful in imaging presynaptic terminals in slices of fixed brain tissue, which are typically difficult to label due to a high degree of nonspecific binding of antibodies and reduced antibody permeability (Figure 4; Supporting Information Figure S4, Table S1). Sagittal sections from Unc13a^WT^ and Unc13a^SNAP/SNAP^ mice were labeled with either BG-JF_646_, SBG-JF_646_, BG-SiR-d12 and SBG-SiR-d12, in parallel to immunolabeling with an antibody against the presynaptic marker Synaptophysin, and a nuclear stain (DAPI). We obtained labeling with SBG-SiR-d12 in regions of high synapse density and where Munc13–1 has been shown to be enriched in a Munc13–1-YFP knock-in mouse line,^46^ for example in the cortex, in the hippocampal *Stratum Orients* and *Stratum Radium*, and in the molecular layer of the cerebellum formation. This signal colocalized with the signal arising from the antibody staining of Synaptophysin 1, and little background was evident in WT control sections. The signal to background ratio of intensity measured in slices obtained from Unc13a^SNAP/SNAP^ and WT mice was 2.4 for SBG-SiR-d12, and lower for other dyes tested (BG-JF_646,_ 1.8-fold; SBG-JF_646_, 1.3-fold; BG-SiR-d12, 0.96-fold; Figure S4). We conclude that fixed tissue from Unc13a^SNAP^ mouse brains can be used for the rapid labeling of presynaptic terminals.

**Figure 4:**
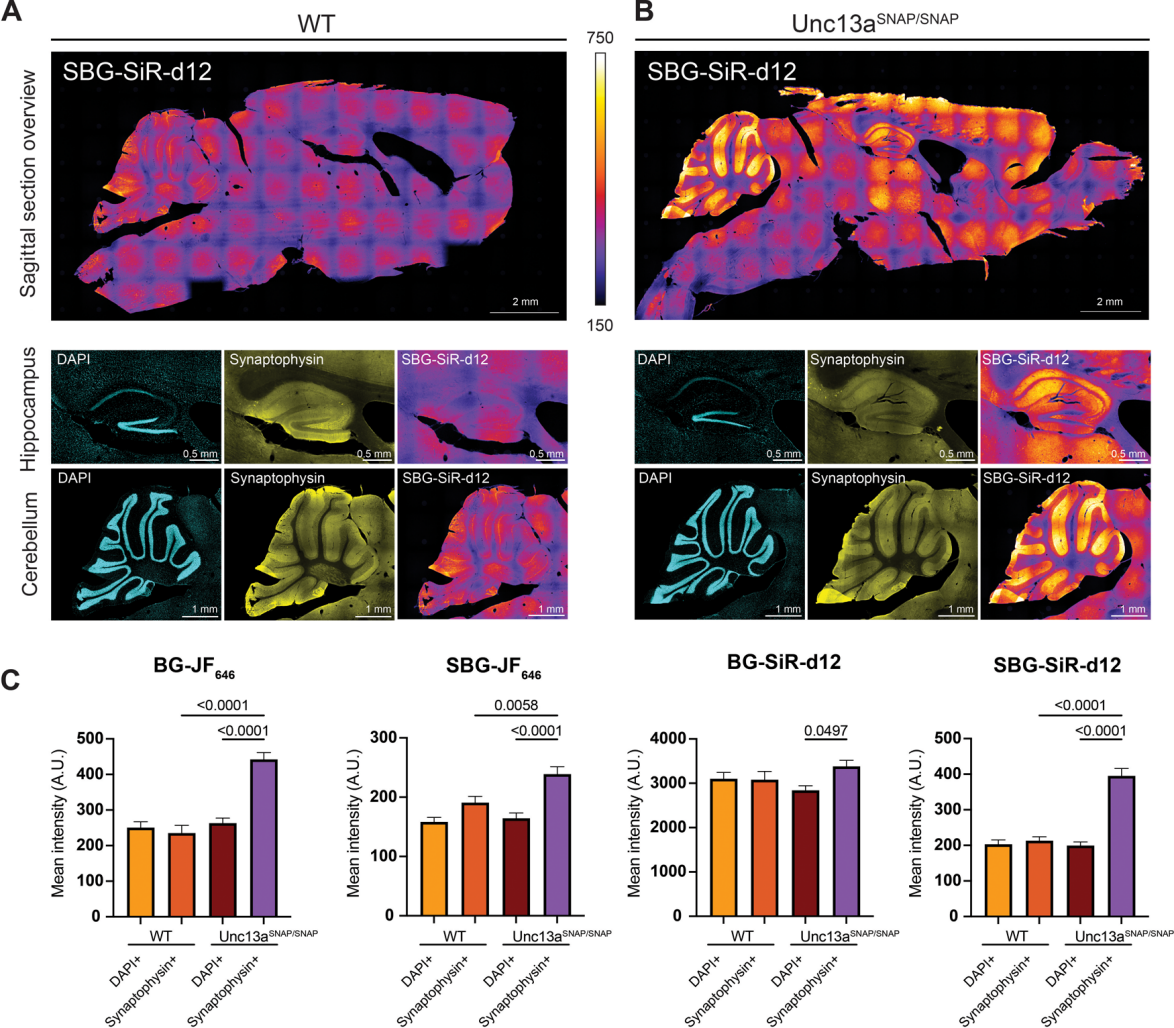
Labeling of presynaptic terminals in brain sections of Unc13a^**SNAP**^ mice. Sagittal brain sections from WT (A) and Unc13a^SNAP/SNAP^ (B) mice were stained with 1 μM SBG-SiR-d12, an antibody against Synaptophysin 1 (yellow; to stain synapses), and with DAPI (cyan; to stain cell nuclei). Example images of the region of the hippocampus (middle) and of the cerebellum (down). (C) Quantification of the SNAP signal overlapping with DAPI or Synaptophysin 1 signals in the cerebellum. The mean intensity of the SNAP signal was measured in randomly selected ROIs containing positive signals for either DAPI (‘DAPI + ’) or Synaptophysin 1 (‘Synaptophysin + ’) (*N* = 2 slices per condition, 20 ROIs for each column and genotype, A.U.: arbitrary units). One-way ANOVA test for multiple comparisons was used to evaluate statistical significance.

### Super-resolution Imaging of Munc13–1-SNAP

Considering the ample data indicating that changes in Munc13–1 nano-organization can underline synaptic plasticity,^36,37,40^ we were interested in establishing whether Munc13–1-SNAP can be imaged at super-resolution, to enable the localization of Munc13–1 beyond the diffraction limit.^10^ We used stimulated emission depletion (STED) nanoscopy and imaged synapses from WT and Unc13a^SNAP/SNAP^ hippocampal neurons in culture, fixed and stained with antibodies against MAP2 (dendrites), Munc13–1, Bassoon, and one of the SNAP-targeting dyes described above. Example images of three synapses per compound are presented in Figure 5A (WT) and 5B (Munc13–1-SNAP) (See also Table S1). Using antibody staining, we identified Munc13–1 puncta that were localized in proximity to the active zone protein Bassoon, and were in part concentrated in nanoclusters. Imaging of Munc13–1 via the SNAP tag resulted in specific signals in Unc13a^SNAP/SNAP^ synapses. As in confocal imaging, we found a high degree of background when labeling WT samples with SBG-JF_646_, and the best signal to background ratio when using SBG-SiR-d12 (Figure 5C; BG-JF_646,_ 4.9-fold; SBG-JF_646_, 1.8-fold; BG-SiR-d12, 7.2-fold; SBG-SiR-d12, 9.6-fold). The SNAP tag signals were colocalized with the signals arising from the Munc13–1 antibody labeling (Figure 5D). Signal intensities, however, were weaker compared to the antibody labeling, which is expected due to the lack of signal amplification by primary and secondary antibodies.

**Figure 5:**
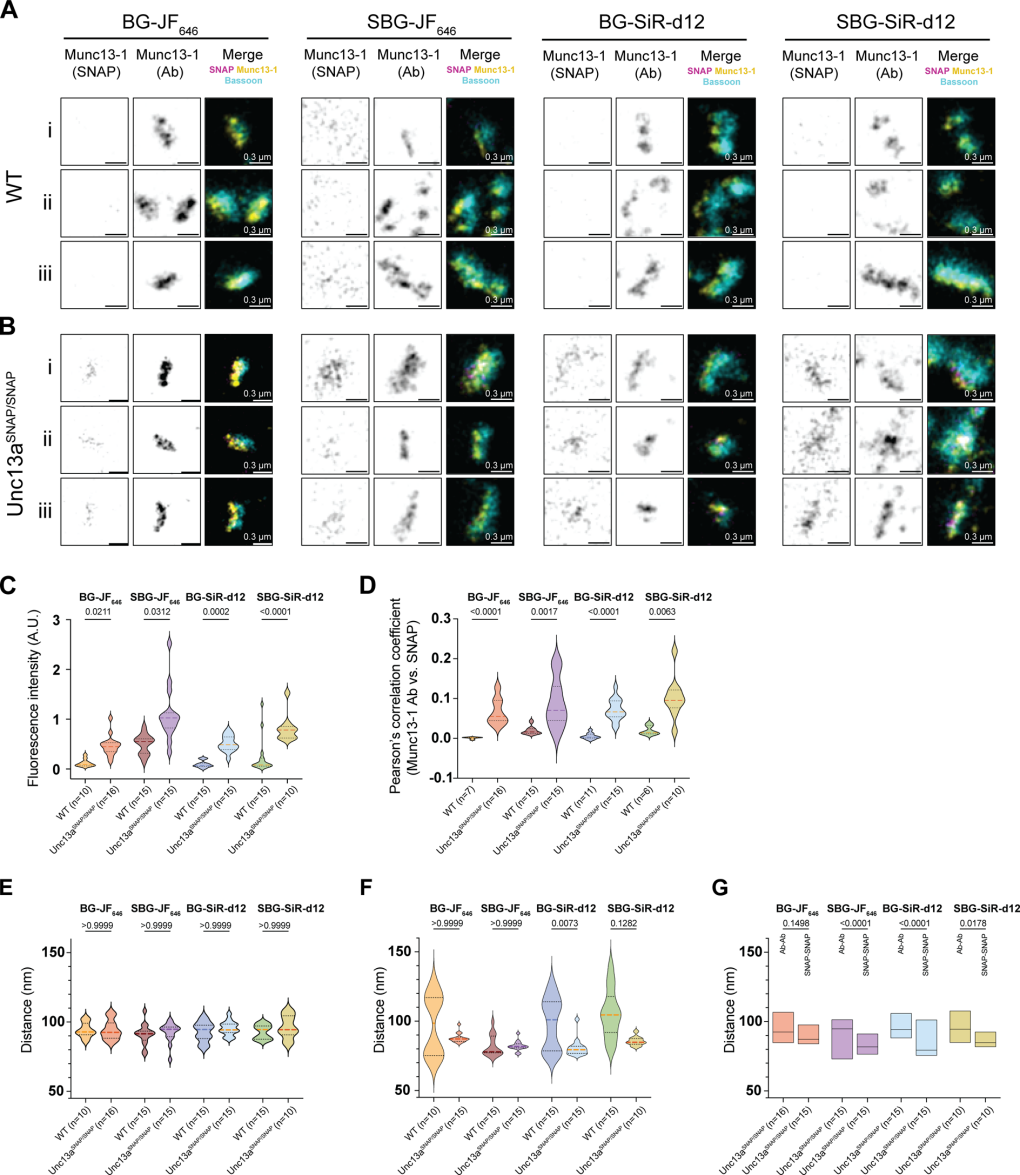
STED microscopy of Munc13–1-SNAP. (A) Example images of synapses from WT and (B) Unc13a^SNAP/SNAP^ neurons labeled with the SNAP tag compounds BG-JF_646_, SBG-JF_646_, BG-SiR-d12, SBG-SiR-d12, as well as with antibodies (Ab) against Munc13–1 and Bassoon, and imaged using STED microscopy. Three synapses are illustrated per condition (Scale bar: 0.3 μm). (C) Quantification of the fluorescence signal intensity arising from Munc13–1-SNAP labeling by BG-JF_646_, SBG-JF_646_, BG-SiR-d12, SBG-SiR-d12, in neurons from WT and Unc13a^SNAP/SNAP^ mice. (D) Colocalization of signals arising from Munc13–1 antibody labeling and SNAP tag labeling using Pearson’s correlation coefficient. (E–G) Munc13–1 puncta-to-puncta distance measured based on (E) antibody labeling or (F) SNAP tag labeling, and (G) a comparison of antibody labeling versus SNAP labeling per SNAP dye tested. In the violin plots, lines represent the median, and 25% and 75% quartiles. In all figures, n represents the number of images analyzed, and a two-tailed Kruskal–Wallis test followed by Dunn’s test for multiple comparisons was used for the analysis of statistical significance. Data were obtained from three cultures. A.U.: arbitrary units.

We then used the images to analyze the distances between Munc13–1 puncta. No differences in puncta-to-puncta distance were observed when comparing WT and Unc13a^SNAP/SNAP^ synapses stained using an antibody against Munc13–1, indicating that Munc13–1 tagging does not disrupt the distribution of Munc13–1 (Figure 5E). The distances between Munc13–1 puncta obtained based on the SNAP tag labeling were broadly distributed in WT synapses, consistent with the background nature of the signal (Figure 5F), whereas a tight distribution was measured in Unc13a^SNAP/SNAP^ neurons. Finally, we compared the puncta-to-puncta distances generated by SNAP tag staining with those generated using antibody staining. We found that distances were significantly shorter when labeled via the SNAP tag across all tested dyes (Figure 5G). These findings suggest that the labeling of Munc13–1 via endogenous SNAP tagging may serve as a valuable complementary approach to antibody labeling.

### Live Labeling and Imaging of Munc13–1-SNAP

One significant advantage of self-labeling protein tags is that they offer the possibility of monitoring proteins in living cells. We therefore tested whether we could utilize Unc13a^SNAP^ neurons for monitoring Munc13–1 using live imaging. In our protocol, WT and Unc13a^SNAP/SNAP^ neurons were incubated with 0.1 μM BG-SiR-d12 or BG-JF_646_ dissolved in a physiological buffer for 45–60 min, and were then allowed to recover for 24–48 h in growth media before subjected to imaging (Figure 6A). The half-life of Munc13–1 has been estimated to be in the range of 3.5–4 days in neuronal cultures,^53,54^ which is compatible with such long recovery times. Very short recovery times resulted in a significant level of background staining, but we did not test the intermediate recovery times. The neurons were then subjected to live cell imaging on a STED microscope with 640 nm excitation and 775 nm depletion at 37 °C. In confocal mode, we observed strong punctate staining that was highly specific for Unc13a^SNAP/SNAP^ neurons, and merely absent in WT neurons (Figure 6B). We then acquired images at STED resolution and readily observed Munc13–1 nanoclusters (Figure 6C). To ascertain that these signals largely represent synapses, we conducted a second experiment where we used lentiviral-mediated transduction to sparsely express a GFP-tagged vesicular glutamate transporter (VGLUT1), a presynaptic marker, in Unc13a^SNAP/SNAP^ neurons (Figure 6D). We observed GFP and BG-SiR-d12 puncta in close apposition. Subjecting the samples to STED imaging, we again observed Munc13–1 clusters colocalizing with the GFP label (Figure 6D lower and right panel). Finally, we quantified the Unc13a nanocluster diameter and found a median diameter of 135–160 nm. This diameter is larger than that described by others,^40^ reflecting the limited labeling density and an estimated resolution of 60 nm for live STED in our setup. We conclude that the Unc13a^SNAP^ mice enable stable and bright live imaging of presynaptic terminals at confocal- and super-resolution.

**Figure 6:**
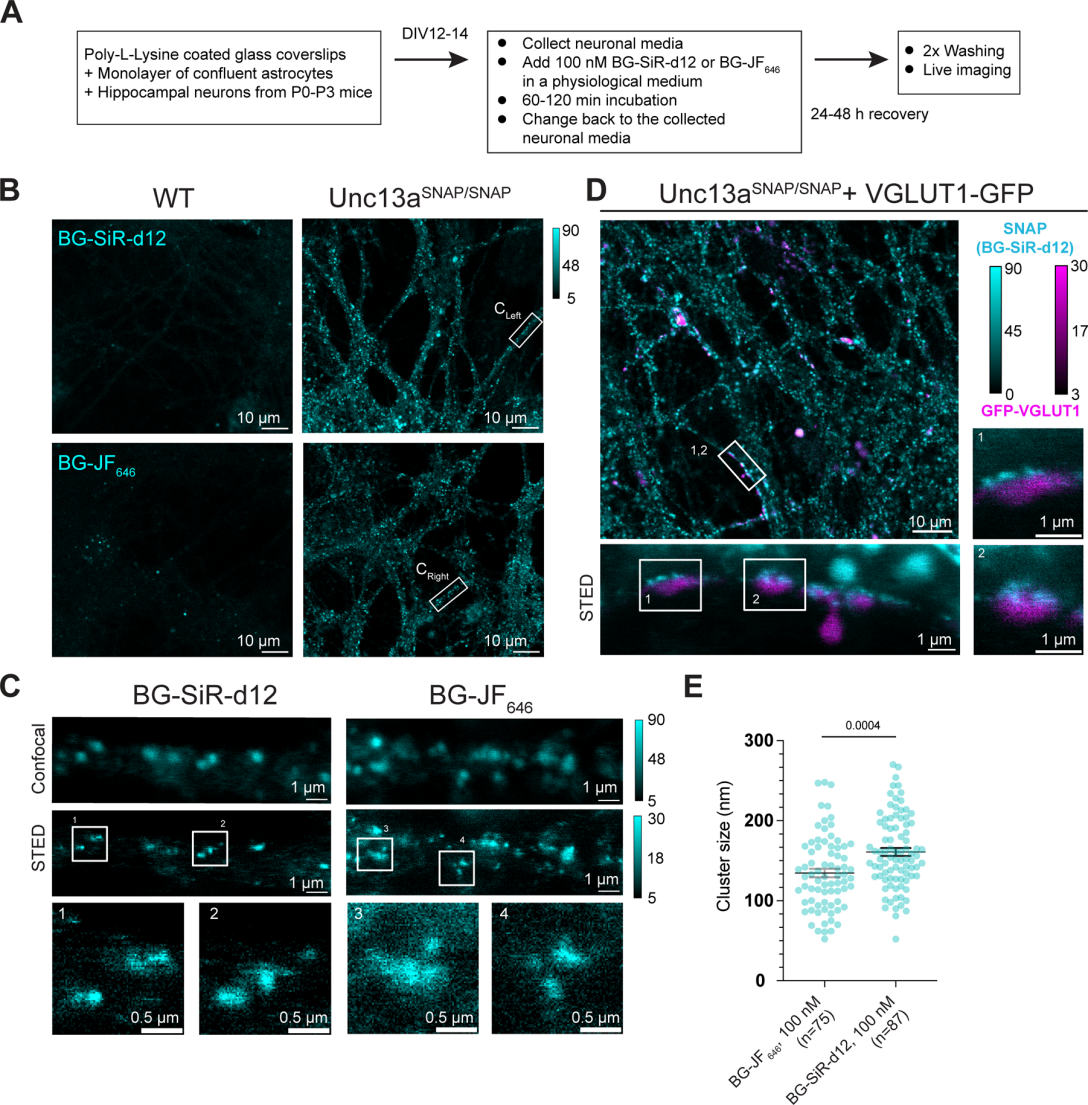
Live imaging of cultured neurons from Unc13a^SNAP^mice. (A) Labeling protocol established for live imaging of Munc13–1-SNAP using a Nikon Eclipse TI microscope with a STEDYCON system. (B) Example images at confocal resolution of live WT (left) and Unc13a^SNAP/SNAP^ neurons (right) stained with BG-SiR-d12 (up) or BG-JF_646_ (down), and (C) Confocal and STED imaging of selected processes from the images in (B). (D) Example images of Unc13a^SNAP/SNAP^ neurons that were infected with a lentivirus expressing a GFP-tagged vesicular glutamate transporter (VGLUT1-GFP; magenta), stained with BG-SiR-d12 (cyan), and imaged at confocal (up) and STED resolution (down and right). (E) Quantification of the Munc13–1 nanocluster diameter using line scans and Gaussian fit in images obtained during live STED imaging. In Figure 6E, data from individual puncta is plotted as mean ± SEM, Mann–Whitney test for statistical significance.

## Discussion

Changes in synaptic function have been repeatedly linked to changes in the organization and composition of the neurotransmitter release machinery. At the presynaptic compartment, the proximity (coupling) of release sites to voltage-gated Ca^2+^ channels determines the strength and probability of neurotransmitter release, and it can be dynamically modulated following plasticity-inducing triggers.^55–58^ Plasticity also evokes changes in the size of the active zone,^59^ and in the abundance of key synaptic proteins.^36,38,60,53^ Munc13–1 is a central presynaptic protein that is absolutely essential for neurotransmitter release, and changes in Munc13–1 abundance and nanoarchitecture are emerging as critical for setting neurotransmitter release properties.^40,61^ However, tools are still lacking to monitor such nanoarchitectural changes for endogenous Munc13–1 *in vivo* and *in vitro*.

We used CRISPR-Cas9 to introduce the SNAP tag cassette in the last exon and prior to the stop codon of the *Unc13a* gene. We used the SNAP tag to complement already-available tools for synapse nanoscopy, in particular the PSD95-HaloTag line^62^ that has proven as highly useful for visualizing the postsynaptic density at super-resolution,^63^ and for imaging of synapses in brain slices.^62^ Successful targeting in mouse embryos enabled us to establish a mouse line constitutively expressing Munc13–1-SNAP at endogenous expression levels. In hippocampal neuronal cultures, Munc13–1-SNAP is expressed at WT levels and localizes to the active zone. Fixed neuronal culture samples and brain slices could be labeled via SNAP tag ligands and were successfully imaged via confocal and/or STED microscopy. Importantly, we demonstrated efficient and specific labeling of Munc13-1-SNAP in live neurons in culture, which opens the door for studies where the labeling of Munc13–1 is carried at a time point of choice, to monitor its dynamics in living cells. Together, we validate the Unc13a^SNAP^ mice for future studies of synapse and active zone nanoarchitecture.

The key advantages of self-labeling tags include great flexibility in dye selection and a large and consistently growing toolbox of chemicals available for use. Importantly, reagents do not only differ in the wavelength of the fluorophore attached to them, but also in their chemical and biological properties, e.g., they undergo uptake to different extents and have variable solubilities. Nonetheless, a large fraction of reported investigations using self-labeling tags rely on the application of one derivative only. We show here by testing several BG derivatives simultaneously that the performance of different BG-fluorophore compounds is assay-dependent and needs to be carefully controlled.^3,64^ Moreover, we added SBG-SiR-d12 as an additional compound in the SNAP dye toolbox. While a priori using a sulfonated and impermeable dye seems counterintuitive, we speculated that SBG dyes will be functional in the context of fixed and permeabilized samples. SBG-SiR-d12 outperformed other conjugates in signal strength and had the highest signal to background ratio in fixed cell (10.6-fold) and in complex brain tissue samples (2.4-fold). Its enhanced performance with respect to SBG-JF_646_ could be attributed to a lower pH sensitivity and to a lower degree of unspecific protein binding, which may result in a lower background signal. We propose SNAP labeling in fixed cell cultures and slices with SBG-SiR-d12 as an alternative to membrane-permeable SNAP dyes. The protocol presented here joins protocols for SNAP tag labeling in the live setting, e.g. in immortalized cell lines,^3^ *Drosophila* brain,^65,66^ in mice for neural identification and ablation,^67^ for receptor localization and optical manipulation,^68^ and for pancreatic islets labeling.^69^

Further developments of imaging configurations, SNAP tag reagents, and labeling protocols, are expected to expand the range of experimentation possible using the Unc13a^SNAP^ mice.^70–74^ The genetic fusion via CRISPR/Cas9 ensures the visualization of Munc13–1 at endogenous expression patterns and levels, independent of constraints related to antibody species, and in the context of neuronal networks in either fixed or live preparations. These key features are expected to make the Unc13a^SNAP^ mice useful in the discovery of synaptic nanoarchitectural principles in the future.

## Materials and Methods

### General

BG-JF_646_, SBG-JF_646_, and BG-SiR-d12 have been synthesized and used previously in our laboratories.^10,42^

### Chemical Synthesis

SBG-SiR-d12 was synthesized as follows: In an Eppendorf tube 1.2 mg (2.0 μmol, 1.0 equiv) of SiR-d12-COOH^45^ was dissolved in 100 μL/mg DMF and 8.0 equiv of DIPEA. Upon addition of 2.3 equiv. TSTU (from a 41 mg/mL stock in DMSO) the reaction mixture was vortexed and allowed to incubate for 10 min, before 1.2 equiv of SBG-NH_2_ ^13^ was added. The mixture was vortexed again and allowed to incubate for 60 min before it was quenched by addition of 20 equiv of acetic acid and 25 vol % of water. C18 RP-HPLC (MeCN:H_2_O+0.1% TFA = 10:90 to 90:10 over 45 min) provided the desired compound, SBG-SiR-d12, which was obtained as a blue powder after lyophilization in 42% yield (0.84 μmol) and aliquoted to 5 nmol to be stored at -20 °C.

^1^H NMR (600 MHz, MeOD-*d*_4_): δ [ppm] = 9.29–9.21 (m, 1H), 8.23–8.16 (m, 1H), 8.12–8.08 (m, 1H), 7.71 (s, 1H), 7.50 (d, *J* = 7.9 Hz, 2H), 7.39 (d, *J* = 7.9 Hz, 2H), 7.20 (s, 2H), 6.86 (d, *J* = 9.1 Hz, 2H), 6.70 (dd, *J* = 9.3, 3.0 Hz, 2H), 5.59 (s, 2H), 4.59–4.55 (m, 2H), 3.54 (t, *J* = 6.5 Hz, 2H), 3.45–3.41 (m, 1H), 3.20–3.18 (m, 1H), 3.12 (t, *J* = 7.0 Hz, 2H), 2.90 (t, *J* = 6.5 Hz, 2H), 2.70 (t, *J* = 7.0 Hz, 2H), 0.63 (s, 3H), 0.57 (s, 3H).

HRMS (ESI): calcd. for C_45_H_38_D_12_N_9_O_8_SSi [M + H]^+^: 916.4020, found: 916.4165.

Raw fluorescence and fluorescence polarization measurements were performed on a TECAN Spark Cyto (λ_Ex_ = 610 ± 20 nm; λ_Em_ = 650 ± 9 nm) and on a TECAN GENios Pro plate reader (λ_Ex_ = 610 ± 15 nm; λ_Em_ = 650 ± 20 nm), respectively. Stocks of substrates (100 nM) were prepared in PBS with either adjusted pH or additional BSA as indicated in a Greiner black flat bottom 96 well plate. Experiments were run in replicates and plotted in Prism 10.

### Animal Study Approval

The generation and use of the Unc13a^SNAP^ (Unc13a^em1(SNAP)Bros^) knock-in mice were approved by the responsible local government organization (Niedersächsisches Landesamt für Verbraucherschutz and Lebensmittelsicherheit; 33.9–42502–04–13/1359 and 33.19- 33.19–42502–04–20/3589). All the experiments performed in Berlin complied with European law and the state of Berlin animal welfare body (LAGeSo).

### Generation of the Unc13a^SNAP^ Knock-In Mouse Line

Superovulated C57BL6/J females were mated with C57BL6/J males, and fertilized eggs were collected. In-house prepared CRISPR reagents, including the hCas9_mRNA, sgRNAs, preformed Cas9_sgRNA RNP complexes, and the dsDNA used as a repair template (HDR fragment) were microinjected into the pronucleus and the cytoplasm of zygotes at the pronuclear stage using an Eppendorf Femtojet. Importantly, all nucleotide-based CRISPR-Cas9 reagents (sgRNAs and hCAS9_mRNA) were used as RNA molecules and were not plasmid-encoded, reducing the probability of off-target effects, due to the short life-time of RNA-based reagents.^75,76^ The sgRNAs targeting the region around the Munc13–1 STOP-codon were selected using the guide RNA selection tool CRISPOR.^77,78^ The correct site-specific insertion of the HDR fragment was confirmed by two localization PCRs with primers upstream and downstream of the HDR sequence followed by direct sequencing of the obtained PCR products. The sequences of the RNA and DNA fragments used to generate and validate the mice are available in Supporting Information. We term the line as Unc13a^em1(SNAP)Bros^. For questions about the usage of the line, please contact Nils Brose and Noa Lipstein.

### Synaptosome Preparation and Western Blot Analysis

A crude synaptosomal fraction (P2) was prepared as described in^79^ from mice age 7–11 weeks. The sample was solubilized to a final concentration of 2 mg/mL in 50 mM Tris/HCl pH 8, 150 mM NaCl, 1 mM CaCl_2_, 1 mM EGTA, 1% NP-40, 0.2 mM phenylmethylsulfonyl fluoride, 1 mg/mL aprotinin, and 0.5 mg/mL leupeptin, stirred on ice for at least 15 min at 4 °C, centrifuged at 4 °C, 20,000g for 5 min to remove insoluble material, mixed with Laemmli sample buffer, and boiled for 10 min at 99 °C. Five μg of each sample were loaded on a 4–12% Bis-Tris gel, and the proteins were separated and transferred to a nitrocellulose membrane. The membrane was washed three times with ddH_2_O, stained with Pierce Reversible Protein Stain Kit for Nitrocellulose Membranes (Thermo Scientific 24580) and scanned. Then, the membrane was blocked for 60 min with blocking buffer (5% (*w*/*v*) low fat milk in Tris buffered Saline (TBS) buffer supplemented with 0.1% Tween20 (TBS-T)), and blotted with the following antibodies diluted in blocking buffer for 2 h at RT with gentle shaking: Rabbit anti Munc13–1 (Synaptic Systems 126 103, diluted 1:1000), Rabbit anti SNAP (New England Biolabs, P9310S, diluted 1:500), Mouse anti synapsin 1 (Synaptic Systems 106 011, diluted 1:1000), Mouse anti Syntaxin 1A/B (Synaptic Systems 110 011, diluted 1:2000), Rabbit anti Rim1 (Synaptic Systems 140 003, diluted 1:1000). The membrane was washed three times for 10 min each with TBS-T before it was blotted for 1 h at RT with secondary antibodies conjugated with horseradish peroxidase (HRP), diluted in blocking buffer: Goat-anti-Rabbit-HRP (Jackson immune 111–035–114, diluted 1:5000) and Goat-anti-Mouse-HRP (Jackson immuno 115–035–146, diluted 1:5000). Before developing the chemiluminescence signal using the standard HRP signal amplification system, the membrane was washed three times for 10 min each in TBS-T buffer and once shortly in TBS buffer.

### HEK293T Cultures

HEK293T cells were cultured in growth medium (DMEM, Glutamax, 4.5 g of glucose, 10% FCS, 1% PS; Invitrogen) at 37 °C and 5% CO_2_. 30,000 cells per well were seeded on 8-well μL slides (Ibidi) previously coated with 0.25 mg/mL poly-l-lysine (Aldrich, mol wt 70 000–150 000). The next day, 400 ng DNA was transfected using 0.8 μL Jet Prime reagent in 40 μL Jet Prime buffer (VWR) per well. Medium was exchanged against antibiotic-free media before the transfection mix was pipetted on the cells. After 4 h incubation at 37 °C and 5% CO_2_, the medium was exchanged against growth media. After 24 h, the cells were stained and imaged. All dyes were used at a concentration of 500 nM. Five μM Hoechst 33342 was used to stain DNA. The staining was conducted in growth medium at 37 °C, 5% CO_2_ for 30 min. Afterward, the cells were washed once in growth media and imaged live in cell imaging buffer (Invitrogen).

### Neuronal Cultures

Primary hippocampal neurons were cultured as previously described.^80^ Briefly, hippocampi were dissected from Unc13a^SNAP/SNAP^, Unc13a^WT/SNAP^, and Unc13a^WT/WT^ P0–P1 littermate mice, and were incubated at 37 °C gently shaking in a solution containing 0.2 mg/mL l-cysteine, 1 mM CaCl_2_, 0.5 mM ethylenediaminetetraacetic acid (EDTA) and 25 units/ml of papain (Worthington Biochemicals), pH 8 in Dulbecco’s modified eagle medium. After 45 min, the solution was replaced by a prewarmed solution containing 2.5 mg/mL Bovine Serum Albumin, 2.5 mg/mL trypsin inhibitor, 1% Fetal Bovine Serum (heat inactivated) in Dulbecco’s modified eagle medium, and the hippocampi were incubated for 15 min at 37 °C gently shaking. The solution was replaced by neuronal culture medium (Neurobasal-A medium supplemented with 2% B-27 Plus Supplement, 1% GlutaMAX supplement, and 0.2% Penicillin-Streptomycin), and the hippocampi were gently triturated to produce a cell suspension that was plated on PLL-coated coverslips for 2–3 weeks at 37 °C and 5% CO_2_. The culture contains primarily neurons and a few astrocytes. Neurons were used for experiments at day-in vitro 14–17.

### Lentiviral Preparation

The construct encoding VGLUT1-GFP was a gift from C. Rosenmund and is based on the FUGW vector,^81^ in which the ubiquitin promoter was exchanged by the human synapsin 1 promoter. Lentiviral preparation was carried out by the viral core facility of the Charite - Universitätsmedizin Berlin (vcf.charite.de) according to the protocol published in^81^ and modified as in,^82^ using the helper plasmids provided by addgene # 8454 and # 8455.^83^

### Electrophysiology

Neurons were prepared from brains of P1 Unc13a^WT^ and Unc13a^SNAP/SNAP^ littermate mice, plated on WT astrocyte microisland cultures according to published protocols,^80^ and kept at 37 °C, 5% CO_2_ until recordings were made at DIV12–14. Whole-cell voltage-clamp data were acquired by using a HEKA EPC10 USB amplifier and the PATCHMASTER software (Molecular Devices). All recordings were performed using an external solution containing 140 mM NaCl, 2.4 mM KCl, 10 mM HEPES, 10 mM glucose, 4 mM CaCl_2_, and 4 mM MgCl_2_ (320 mOsm/l). The standard internal solution contained 136 mM KCl, 17.8 mM HEPES, 1 mM EGTA, 4.6 mM MgCl_2_, 4 mM NaATP, 0.3 mM Na_2_GTP, 15 mM creatine phosphate, and 5 U/ml phosphocreatine kinase (315–320 mOsm/l), pH 7.4. Recordings were made at room temperature (∼22 °C). eEPSCs were evoked by depolarizing the cell from – 70 to 0 mV for a 1 ms duration. Basal eEPSCs were recorded at a frequency of 0.1 Hz. mEPSCs were recorded for 50 s in the absence of tetrodotoxin. mEPSCs traces were filtered at 1 kHz and miniature events were identified using a sliding template function. Analyses were performed using an Axograph 1.4.3 (MolecularDevices) or Igor Pro (Wavemetrics). Electrophysiological data are presented as mean ± SEM.

### Immunocytochemical Staining Protocols

Experiments were made in neuronal cultures at days-in vitro 14–17. Neurons were washed twice with PBS, and fixed by adding cold 1% paraformaldehyde for 10 min on ice. Neurons were again washed and leftover paraformaldehyde was quenched by adding 50 mM cold glycine for 10 min, followed by two additional washes with PBS. The cells were either stored in PBS at 4 °C or immediately used for immunostaining. The cell membrane was permeabilized with cold 0.25% Triton-X-100 in PBS for 10 min and rinsed once in pure PBS. To block nonspecific binding sites, the cells were incubated in cold 0.3% NGS in PBS for 20 min. All steps were performed with the multiwell plate stored on ice to prevent protein degradation.

To label Munc13–1 using the SNAP tag, 100-250 nmol BG-dye conjugates were diluted in blocking solution and applied to the neurons for 30 min at room temperature (RT) in the dark. Subsequently, the cells were extensively washed up to eight times in blocking solution, and the following antibodies were applied in blocking buffer for immunolabeling: Chicken polyclonal anti MAP2 (Novus Biologicals NB300–213, diluted 1:1000), Rabbit polyclonal anti Munc13–1 (Synaptic Systems AB_126103, diluted 1:500), and Guinea Pig polyclonal anti Bassoon (Synaptic Systems AB_141004, diluted 1:500). Incubation was performed for 1 h at RT in the dark. To remove unbound antibodies, the cultures were washed three times with blocking solution before applying the following secondary antibodies conjugated with fluorophores: Anti-Chicken-405, Anti-Guinea Pig-488 and Anti-Rabbit-594 (all diluted 1:500). The neurons were incubated with the secondary antibodies for 30 min at RT in the dark and washed eight times in PBS to remove unbound products. The coverslips on which the neurons were fixed were mounted with ProLong Gold Antifade Mountant (Invitrogen P36934) on microscope glass slides and stored in the dark at RT for 48 h until dry before imaging.

### Brain Sections and Immunohistochemistry

Animals were transcardially perfused with fixative (4% paraformaldehyde in 1X PBS, pH 7.4). Brains were postfixed overnight at 4 °C, incubated in 30% sucrose in 1X PBS for a minimum of 48 h, and snap frozen with 2-methylbutan on dry ice. Tissues were sectioned at 30 μm using a cryostat. Floating sections were kept in 30% ethylene glycol and 30% glycerol in 1X PBS at −20 °C. For immunostaining, sections were washed three times in 1X PBS before incubation with a blocking solution containing 10% normal donkey serum (NDS) in TBS-T (0.05% Triton X-100 in 1X TBS). The slices were incubated with one of the SNAP tag dyes (1 μM) and anti-Synaptophysin1 (Synaptic Systems AB_101002, diluted 1:200) at 4 °C overnight in TBS-T containing 2% NDS. Sections were washed three times in 1X TBS-T, followed by incubation for 2 h at room temperature in the dark with secondary antibody (Alexa Fluor 488-AffiniPure Donkey Anti-Rabbit IgG, Jackson Research 711–545–152, diluted 1:1000,) and DAPI (Sigma MBD0015–1 ML, diluted 1:10000). The sections were washed three times in 1X TBS-T before mounting onto microscope slides with ProLong Glass antifade (InvitrogenP36982).

### Confocal and STED Imaging

Confocal and STED microscopy on fixed neuronal samples was performed using a Leica SP8 TCS STED FALCON (Leica Microsystems). Confocal images were collected using a time gated Hybrid detector (0.5–6 ns). Images of 1024 × 1024 pixel had a pixel size of 130 nm for confocal imaging and 20 nm for STED imaging. For imaging far red SNAP dyes, we used the following settings: excitation wavelength 646 nm, emission wavelength 656–751 nm. Brain sections were imaged on a Nikon Spinning Disk Field Scanning Confocal System using a 10x objective (Plan Apo λD, NA 0.45), acquired in the “Large Image” mode of NIS 5.4 Software and stitched with 20% overlap between adjacent frames. For imaging DAPI, Synaptophysin 1 and SNAP dyes 405, 488, and 640 nm excitation lasers and 440/40, 525/50, 708/75 emission filters were used, respectively. CA-JF_519_ and SBG-SiR-d12 in live transfected HEK293Ts cells was imaged on a TIE Nikon epifluorescence microscope equipped with a pE4000 (cool LED), Penta Cube (AHF 66–615), 60X oil NA 1.49 (Apo TIRF Nikon) and a sCMOS camera (Prime 95B, Photometrics) operated by NIS Elements (Nikon). For excitation, the following LED wavelengths were used: Hoechst: 405 nm; JF_519_: 480 nm; SiR-d12: 635 nm.

### Live Imaging

Live confocal and STED imaging was done on a STEDYCON system (Abberior Instruments GmbH, Göttingen), mounted on a Nikon Eclipse TI research microscope, equipped with a Plan APO Lambda D100X/1.45 NA oil objective (Nikon) and controlled by NIS Elements (Nikon). Twenty-four h prior to live-cell imaging, an incubation chamber surrounding the microscope setup was set to 37 °C. In order to provide stable focus during imaging, the Perfect Focus System (Nikon) was used. Imaging was performed in a medium containing 140 mM NaCl, 2.4 mM KCl, 10 mM HEPES, 10 mM glucose, 2 mM CaCl_2_, and 2 mM MgCl_2_ (320 mOsm/l) at 37 °C. To capture a SNAP tag signal, excitation was evoked with a 640 nm diode laser, and emission was detected with a single counting avalanche photodiode (650–700 nm). The VGLUT1-GFP signal was generated with excitation using a 488 nm diode laser. A pixel size of 200 nm x 200 or 20 nm × 20 nm were used in confocal and STED imaging, respectively. Confocal images were captured with a line accumulation of 1, while STED images utilized a line accumulation of 5.

### Image Data Analysis

Confocal and STED images (Figures 3, 5 and 6) were analyzed using routines generated in Matlab (The Mathworks Inc., Natick MA, USA; version R2022b). The Bassoon images were subjected to an automated thresholding procedure to identify signals above background (using a threshold equal to the mean intensity value plus the standard deviation, calculated for the entire image). The resulting regions of interest (ROIs) were processed by an erosion procedure, using a kernel of 4 pixels, to remove noise events and only retain synapse-like signals. The remaining ROIs were then automatically dilated to include the surrounding synaptic areas, and the average intensity values were measured and reported for all channels. The analysis sequence was as follows: (1) Thresholding according to the Bassoon signal, thereby obtaining the Bassoon ROIs (synapses); (2) Erosion of the signals above threshold, to remove small objects that represent noise events; (3) Dilation of the remaining ROIs (true synapses), to return them to synapse size; (4) Analysis of intensity in each channel within the respective ROIs; the average background intensity is subtracted from each value. Background is defined as the signal over the entire image, except for within ROIs. (5) Plotting of intensities per ROI or per image and statistical analysis; (6) Pearson’s correlation coefficients across all pixels in a ROI, for each ROI, between the relevant channels (SNAP tag labeling, Munc13 antibody immunostaining); (7) Plotting of correlation coefficients, statistical analysis. The data in Figure 4 was obtained using ImageJ (version 1.54f). Here, (a) the images were Split to 3 channels (1-DAPI, 2-Synaptophysin, 3-SNAP); (b) filtering and segmentation was applied for channels 1 and 2, (c) a mask image was created and 10 ROIs were randomly selected from each mask and from the background; (d) mean intensity for each ROI was measured within the SNAP channel, and (e) the data was subjected to statistical analysis and plotting.

### Statistics

Statistical analysis was conducted in Prism10. For imaging data, experimental groups were compared against each other using the Mann–Whitney test, the Kruskal–Wallis test followed by the Dunn’s test for multiple comparisons, or the ANOVA test. For the Electrophysiological data, statistical significance was determined using the nonparametric Mann–Whitney test.

## Supplementary Material



## Data Availability

The data that support the findings of this study are available upon request from the corresponding author.

## Notes

Generation of an Unc13a^SNAP^ knock-in mouse mutant using CRISPR/Cas9 gene editing, location PCR and genotyping, general chemistry, SBG-SiR-d12 analysis (PDF)

## Abbreviations

Munc13-1: protein unc-13 homologue A
SV: synaptic vesicles
BG: *O* ^6^-benzylguanine
CA: chloroalkane
ALS: amyotrophic lateral sclerosis
FTD: frontotemporal dementia, SiR, silicon rhodamine
WT: wild type
