# Supplementary material for: Endogenous SNAP-Tagging of Munc13‑1 for Monitoring Synapse Nanoarchitecture
Source: JACS Au. 2025 May 23;5(6):2475–90. doi: 10.1021/jacsau.4c00946 (PMC12188394; doi:10.1021/jacsau.4c00946)
Supplement: Supplementary file 1 [file au4c00946_si_001.pdf]

SUPPORTING INFORMATION

Endogenous SNAP-tagging of Munc13-1 for monitoring synapse nanoarchitecture

Maria Kowald[1,‡], Sylvestre P. J. T. Bachollet[1,‡], Fritz Benseler[2], Maria Steinecker[3], Moritz Boll[1], Sofia Kaushik[1], Tolga Soykan[1], Siqi Sun[1], Ramona Birke[1], Dragana Ilic[1], Nils Brose[2], Hanna Hörnberg[3], Martin Lehmann[1], Silvio O. Rizzoli[4,5,6], Johannes Broichhagen[1,*], Noa Lipstein[1,2*]

[1] Leibniz-Forschungsinstitut für Molekulare Pharmakologie (FMP), 13125 Berlin, Germany

[2] Max Planck Institute for Multidisciplinary Sciences, 37075 Göttingen, Germany

[3] Max Delbrück Center for Molecular Medicine in the Helmholtz Association (MDC), 13125 Berlin, Germany

[4] Department of Neuro- and Sensory Physiology, University Medical Center, 37073 Göttingen, Germany

[5] Excellence Cluster Multiscale Bioimaging (MBExC), 37073 Göttingen, Germany

[6] Center for Biostructural Imaging of Neurodegeneration (BIN), University Medical Center Göttingen, 37075 Göttingen, Germany

Corresponding authors: Johannes Broichhagen (Broichhagen@fmp-berlin.de) and Noa Lipstein (Lipstein@fmp-berlin.de)

CONTENT

Supplemental Figures 1-5
Table S1
Generation of an Unc13a^{SNAP} knock-in mouse mutant using CRISPR/Cas9 gene editing
Location PCR and genotyping
General Chemistry
SBG-SiR-d12 analysis

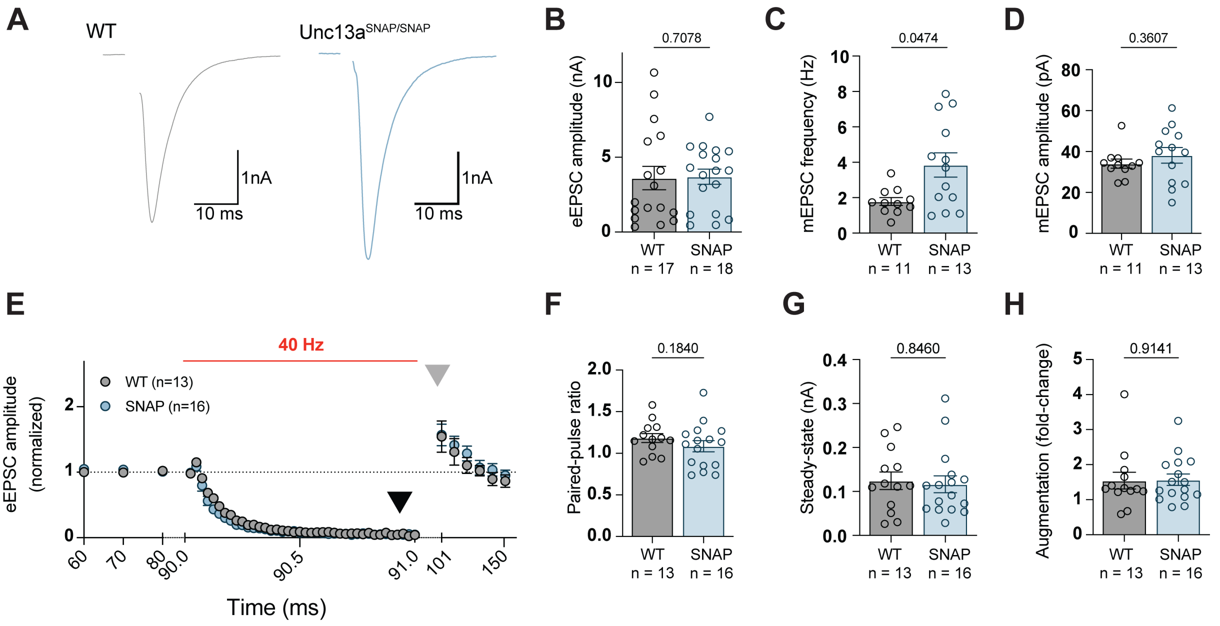

Figure S1: Electrophysiological analysis of synaptic transmission in Unc13a^{SNAP} neurons. (A) Example traces of evoked excitatory postsynaptic currents (eEPSCs) recorded in WT (black) and Munc13-1$^{SNAP/SNAP}$ autaptic hippocampal neurons (light blue), and (**B**) a plot depicting averaged initial eEPSC amplitudes. (**C,D**) Plot depicting the frequency (**C**) and amplitude (**D**) of spontaneous miniature excitatory postsynaptic currents (mEPSCs). (**E**) Averaged and normalized traces of eEPSC amplitudes before, during and after a high frequency action potential train (1 s, 40 Hz). (**F**) Paired-pulse ratios (PPR), calculated as the ratio of the second to the first eEPSC amplitudes ($eEPSC_2/eEPSC_1$) recorded during a 40 Hz action potential train as in (**E**). (**G**) Steady-state eEPSC amplitudes calculated as the average of the last three eEPSC amplitudes recorded during a 40 Hz action potential train (see black arrowhead in E), and (**H**) the normalized eEPSC amplitude evoked by a single action potential and recorded 10 s after the cessation of the 40 Hz train, reflecting augmentation of the synaptic response following high-frequency activity (see grey arrowhead in E). Circles in **B-D**, **F-H** represent values for individual neurons. Data in **E** represents the average of the indicated number of individually-recorded neurons. Data was obtained from two independent cultures. Error bars represent mean ± SEM. Statistical analysis was performed by using a Mann-Whitney test.

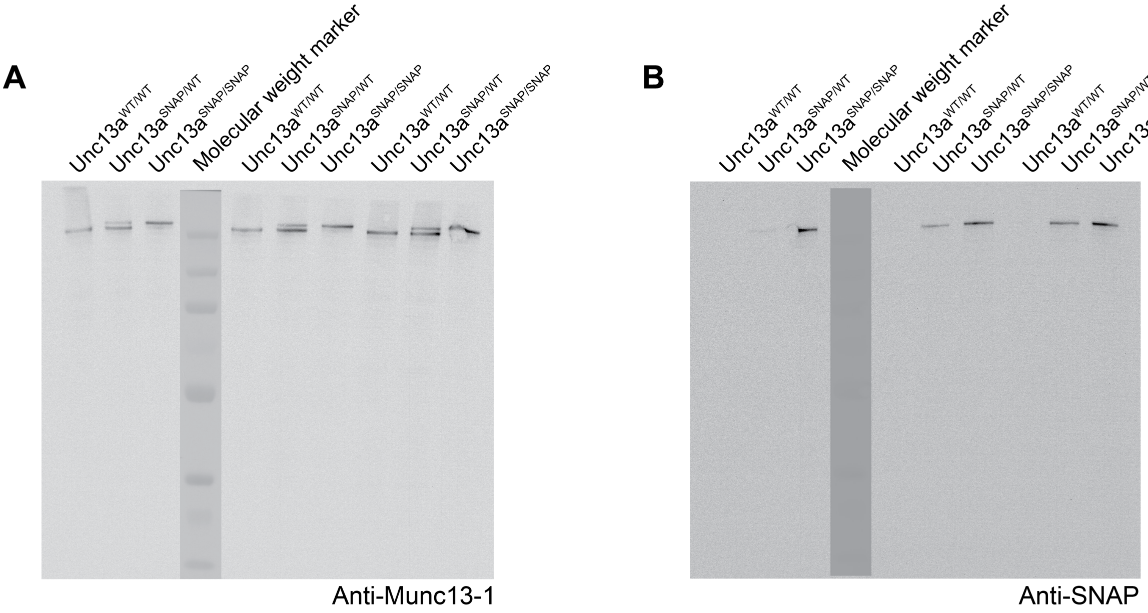

Figure S2: Endogenous tagging of Munc13-1 with a SNAP tag does not result in truncated protein products. Uncut Western blot membranes (used in Figure 1) that were probed with antibodies against **(A)** Munc13-1 or **(B)** the SNAP tag. No truncated protein products are identified in samples from WT, heterozygous and homozygous Unc13a$^{SNAP/SNAP}$ samples.

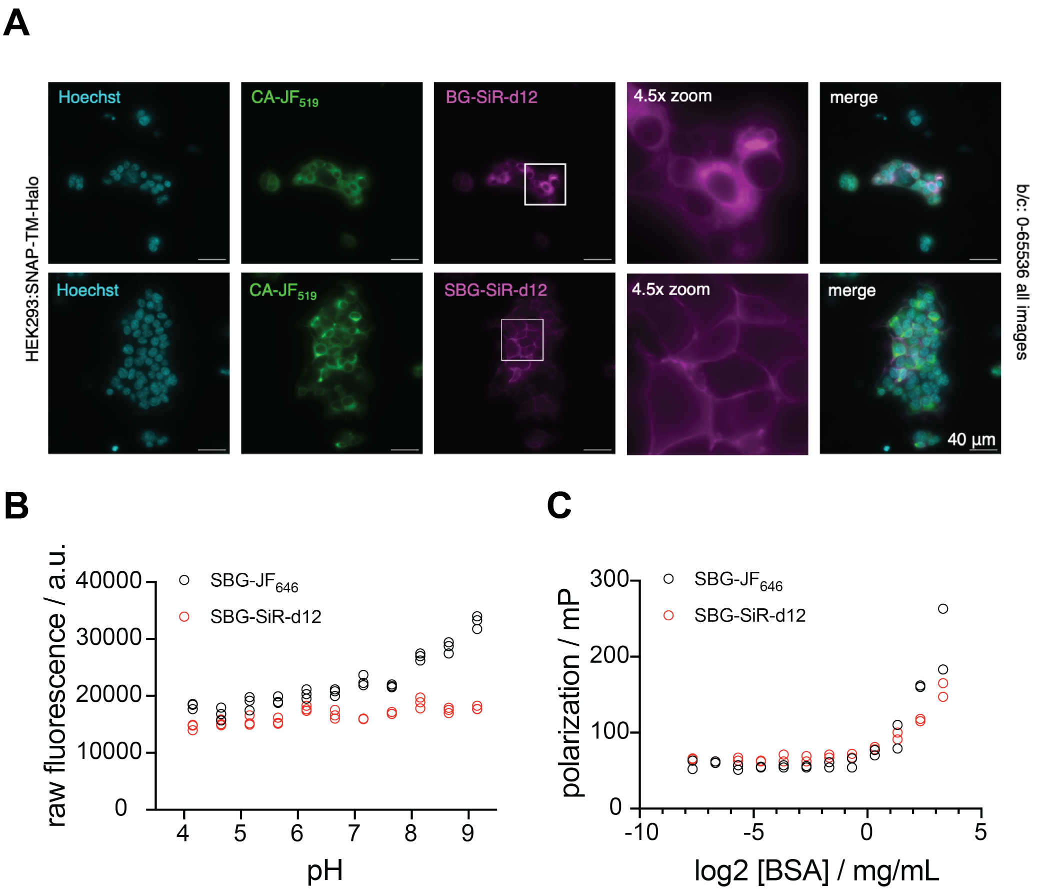

Figure S3: SBG-SiR-d12 is a membrane-impermeable dye. (A) HEK293 cells were transiently transfected with a construct encoding for the expression of SNAP-TM-HTP, a transmembrane construct with an extracellular SNAP tag and an intracellular HaloTag, divided by a single transmembrane helix. Live cells were incubated with CA-JF_{519} targeting the intracellular HaloTag and with either BG-SiR-d12 (upper panel), which can cross membranes (and thus stain intracellular protein pools, for example of newly-translated SNAP-TM-HTP in the endoplasmic reticulum), or with SBG-SiR-d12. Example images of cells with CA-JF_{519} staining clearly demonstrate a diffuse staining by BG-SiR-d12 and a largely membrane-targeted staining by SBG-SiR-d12. White rectangles: indicated areas of zoom-ins. **(B-C)** *In vitro* measurements of SBG-JF_{646} and SBG-SiR-d12. **(B)** pH sensitivity of SBG-JF_{646} and SBG-SiR-d12 (each 100 nM) shows insensitivity for SBG-SiR-d12 and an increase in fluorescence at more basic conditions for SBG-JF_{646} in phosphate buffer. Measurement performed in triplicates. **(C)** Fluorescence polarization to determine the tendency for unspecific binding of bovine serum albumin (BSA) shows a lower tendency for unspecific binding in the case of SBG-SiR-d12 compared to SBG-JF_{646}. Measurement performed in duplicate.

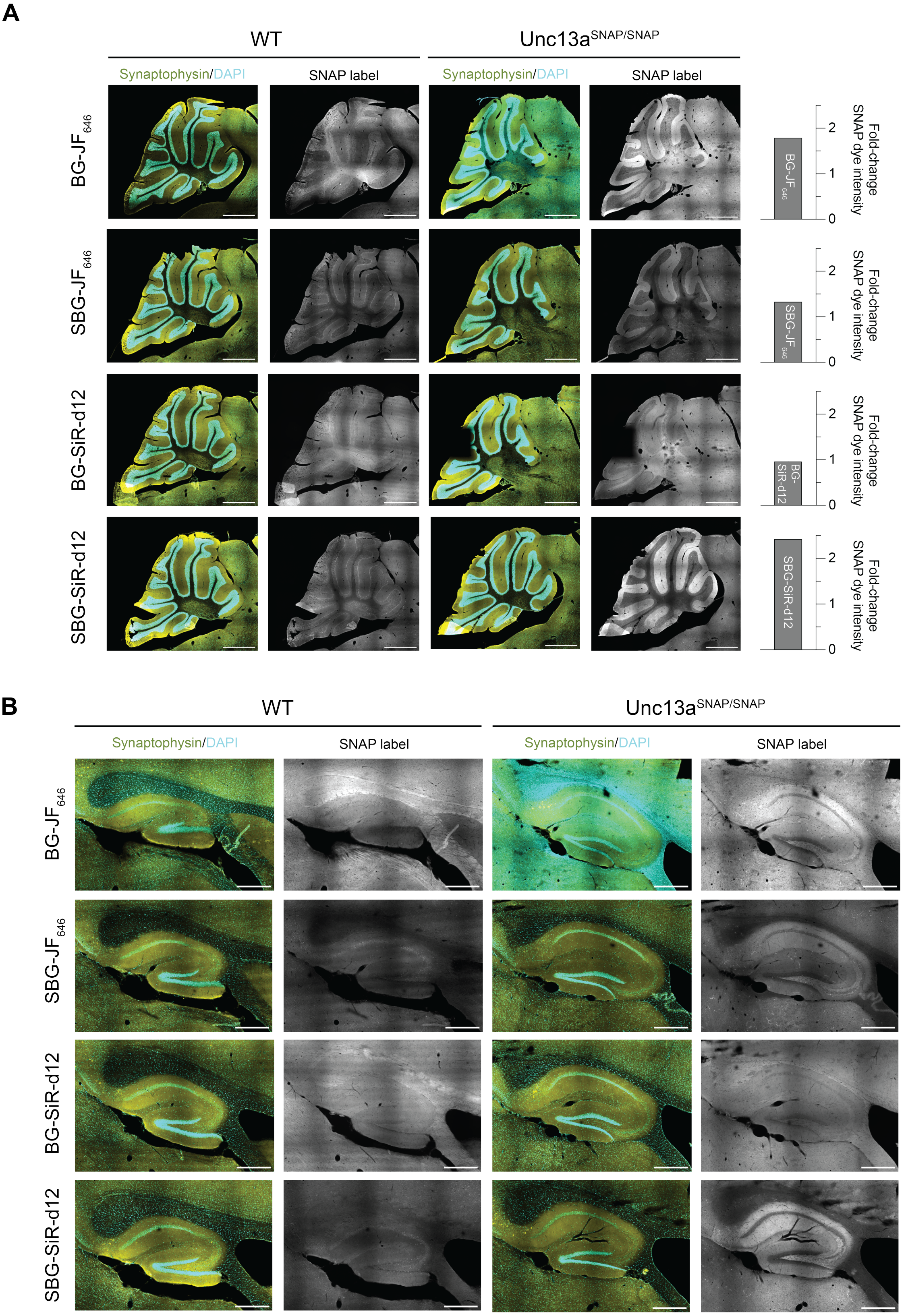

Figure S4: Labeling of presynaptic terminals in brain sections of Unc13a^{SNAP} mice. Sagittal brain sections from WT and Unc13a$^{SNAP/SNAP}$ mice were stained with either of the following SNAP dyes: BG-JF$_{646}$, SBG-JF$_{646}$, BG-SiR-d12, or SBG-SiR-d12, with an antibody against Synaptophysin 1 (green; to stain synapses), and with DAPI (cyan; to stain cell nuclei), as in Figure 4. Example images in the region of the cerebellum (**A**; scale bar 1 mm), and in the region of the hippocampus (**B**; scale bar 0.5 mm).

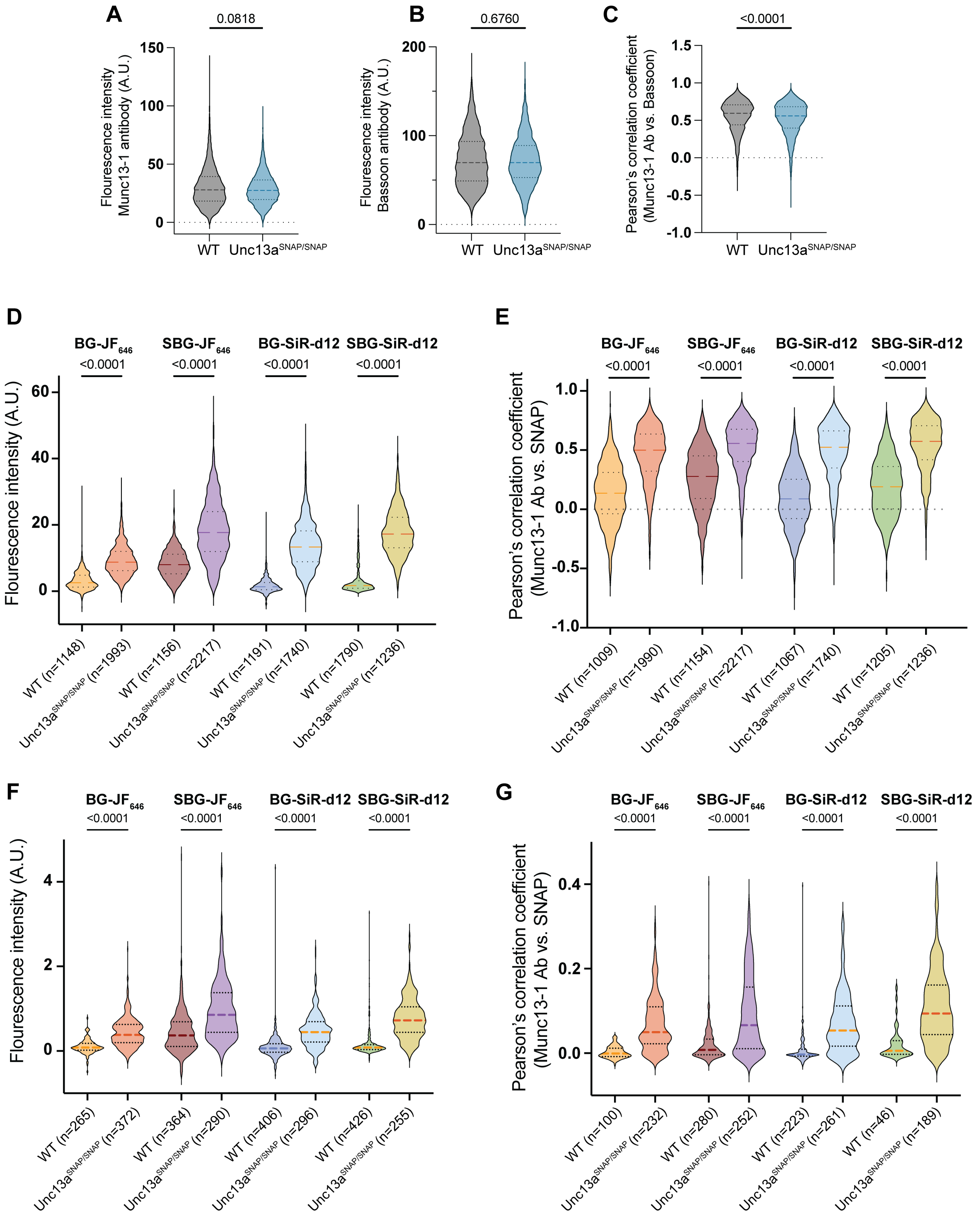

Figure S5: Imaging data plotted according to the number of regions of interest (ROIs). (A-C) Identical data to the data presented in Figure 2 (C-E), plotted according to the number of ROIs. **(D, E)** Identical data to the data presented in Figure 3 (D, E), plotted according to the number of ROIs. **(F,G)** Identical data to the data presented in Figure 5 (C,D), plotted by the number of ROIs. In all violin plots, lines represent the median, 25% and 75% quartiles. Statistical significance was evaluated using a two-tailed Mann-Whitney test (A-C) or a two-sided Kruskal-Wallis test followed by Dunn's test for multiple comparisons (D-G). In each panel, data was obtained from three independent experiments.

Table S1: Summary of parameters used during microscopy experiments

Experiment /Figure	Date	Antigen/Antibody	Dye/ Antibody	Excitation/ Depletion	Laser power*	Detection
Validation of Munc13-1 localization in hippocampal neurons from Unc13a^{SNAP} mice<br>Leica SP8 STED<br>**Fig. 2**<br>$n_{cultures}$ = 3 per condition<br>n_{images} (WT/Unc13a$^{SNAP/SNAP}$) = 55/54<br>n_{ROIs} (WT/Unc13a$^{SNAP/SNAP}$) = 5285/7186	13.6.24<br>17.6.24<br>19.6.24	Munc13-1/<br>RRID:AB_887733<br>1:500	Alexa Fluor™ Plus 594<br>RRID:AB_2762827<br>1:500	Confocal: 594 nm	16%<br>Line Acc. 1	604-654 nm
		Bassoon/<br>RRID:AB_2290619<br>1:500	CF488A<br>RRID:AB_10853117<br>1:500	Confocal: 488 nm	16%<br>Line Acc. 1	498-548 nm
		MAP2/<br>RRID:AB_350528<br>1:1000	Alexa Fluor® 405<br>RRID:AB_2890171<br>1:500	Confocal: 405 nm	100%<br>Line Acc. 1	415-465 nm
Validation of the SNAP tag functionality in cultured, fixed hippocampal neurons<br>Leica SP8 STED<br>**Fig. 3**<br>$n_{cultures}$ = 3 per condition<br>n_{images} = 10-15 per condition<br>n_{ROIs} = 1000-2200<br>See Figure 3 and S5 for precise n values	13.6.24<br>17.6.24<br>19.6.24	Munc13-1/<br>RRID:AB_887733<br>1:500	Alexa Fluor™ Plus 594<br>RRID:AB_2762827<br>1:500	Confocal: 594 nm	16%<br>Line Acc. 1	604-654 nm
		Bassoon/<br>RRID:AB_2290619<br>1:500	CF488A<br>RRID:AB_10853117<br>1:500	Confocal: 488 nm	16%<br>Line Acc. 1	498-548 nm
		MAP2/<br>RRID:AB_350528<br>1:1000	Alexa Fluor® 405<br>RRID:AB_2890171<br>1:500	Confocal: 405 nm	100%<br>Line Acc. 1	415-465 nm
		BG-SiR-d12/<br>SBG-SiR-d12/<br>BG-JF$_{646}$/<br>SBG-JF$_{646}$		Confocal: 647 nm	80%<br>Line Acc. 1	657-707 nm
Labeling of presynaptic terminal in brain sections of Unc13a $^{SNAP/SNAP}$ mice at NIKON TiE CSU-X1 Spinning Disc<br>**Fig. 4**<br>$n_{sections}$ (WT/Unc13a$^{SNAP/SNAP}$) = 2 per condition<br>n_{ROIs}: 20 per condition	13.9.24<br>20.1.25	-	DAPI<br>1:10,000	405 nm	100% (5 mW)	420-460 nm, 100 ms Exp.
		Synaptophysin 1/<br>RRID:AB_887905<br>1:200	CF488A<br>RRID:AB_2313584<br>1:1000	488 nm	100% (7 mW)	500-550 nm<br>500 ms Exp.
		BG-SiR-d12/<br>SBG-SiR-d12/<br>BG-JF$_{646}$/<br>SBG-JF$_{646}$		640 nm	100% (12 mW)	672-744 nm<br>1 s Exp
STED microscopy of Munc13-1-SNAP<br>Leica SP8 STED<br>**Fig. 5**<br>$n_{cultures}$ = 3 per condition<br>n_{images} = 6-16 per condition<br>n_{ROIs} = 46-406<br><br>see Figure 5 and S5 for precise n values	13.6.24<br>17.6.24<br>05.9.24	Munc13-1/<br>RRID:AB_887733<br>1:500	Alexa Fluor™ Plus 594<br>RRID:AB_2762827<br>1:500	STED: 594 nm<br>STED Laser: 775 nm	50%<br>60%<br>Line Acc. 8	656-750 nm
		Bassoon/<br>RRID:AB_2290619<br>1:500	CF488A<br>RRID:AB_10853117<br>1:500	STED: 488 nm<br>STED Laser: 592 nm	40%<br>60%<br>Line Acc. 8	498-550 nm
		BG-SiR-d12/<br>SBG-SiR-d12/<br>BG-JF$_{646}$/<br>SBG-JF$_{646}$	-	STED: 647 nm<br>STED Laser: 775 nm	100%<br>5%<br>Line Acc. 8	656-750 nm
Live Imaging of cultured neurons from Unc13a$^{SNAP/SNAP}$<br>Nikon-TiE StedyCON<br>**Fig. 6**	29.11.24<br>11.12.24<br>17.12.24	BG-SiR-d12/<br>BG-JF$_{646}$	-	Confocal: 640 nm<br><br>STED: 640 nm	100% (51 μW)<br>Line Acc. 1	650-700 nm

$n_{cultures}$ = 3 per condition<br>n_{Images} = 60<br>$n_{nanoclusters}$ (Figure 6E)<br>(Unc13a$^{SNAP/SNAP}$, BG-JF$_{646}$ vs BG-SiR-d12) = 75/87				STED Laser 775 nm	100% (51 µW)<br>Line Acc. 5<br>5% (7 mW)	
		VGLUT1-GFP	-	Confocal: 488 nm	100% (13 µW)<br>Line Acc. 1	500-550 nm

* Laser power for Leica SP8 STED was measured with a 10X air objective at 100% at 240 µW at 405 nm, 180 µW at 488 nm, 800 µW at 640 nm, 320 mW at 592 nm (CW STED depl.) and 320 mW at 775 nm (pulsed STED depl. 500-550 nm; 580-630 nm; 650-700 nm), and behaved linearly.

Generation of an Unc13a^{Snap} knock-in mouse mutant using CRISPR/Cas9 gene editing

Unc13a *sgRNA1&2 protospacer sequences:*
5'- CTGCGCCCTAGCGCGCGTTT-3' (PAM = CGG)
5'- CCGCCACCGAAACGCGCGCT-3' (PAM = AGG)

Munc13-1_Linker_SNAPtag_HDR1_2335bp:

HDR1 sequence:
Unc13a-intronic and 3'-UTR sequence = black lower case
Unc13a -Exon44-CDS = BLACK UPPER CASE
Linker = ORANGE UPPER CASE
SNAP-tag = BLUE UPPER CASE
STOP-codon = RED UPPER CASE
3'UTR point mutations = BOLD UPPER CASE UNDERLINED

5'-
ctgctccggccaatagctttgtcatttagagtagttggctcgagtgtggtgcagggtagggatggccgccatacgcggcagaggtg
ggtactggggagatgggatgatagtcgggggctacaggaggctgactggcgtggcccgatcccaggaaactgagcagaagg
cagggtgcagcagggccgggaatggggcgtggccagaatgggtggaaggagtgggcgtggccggagcccaaactcaagct
gctgcgccttaaggatctcagctgcgctgtggggttcacttagtcgcctgccctacgggatgcgctgggcagatcgggtctgggctg
tgggtttcctagtgggcgtggcctagttggagggctttttctgggcgcaacctgggtagcgtggaaggggttctctagccaactttcta
ggagtacgaactactaacgtatgtggcggtgtggtgtgaaccgctcgctggtagctcccctgcgtgacggcgggtgactgggctc
acggtggcctctcgctgagccggtccttgcccacagCTCCCTGAGCGCCGACGCGGGCCCCGAGTGCTA
CGAGCTGCAGGTGTGCGTGAAGGACTACTGCTTTGCGCGCGAAGACCGCACGGTGGG
GCTGGCGGTGCTGCAACTGCGAGAGCTGGCCCAGCGCGGAGCGCCGCGTGCTGGC
TTCCACTCGGCCGCCGCATCCACATGGACGACACGGGGCTCACAGTGCTGCGCATCCT
GTCGCAGCGCAGCAACGATGAGGTGGCCAAGGAATTCGTCAAGCTCAAGTCGGACACG
CGCTCAGCCGAGGAGGGCGGTGCCGCGCCTGCGCCCGGTGGAAGCGGCGGAAGCGG
CGGTAGCATGGACAAAGACTGCGAAATGAAGCGCACCACCCTGGATAGCCCTCTGGGC
AAGCTGGAACTGTCTGGGTGCGAACAGGGCCTGCACCGTATCATCTTCCTGGGCAAAG
GAACATCTGCCGCCGACGCCGTGGAAGTGCCTGCCCCAGCCGCCGTGCTGGGCGGAC
CAGAGCCACTGATGCAGGCCACCGCCTGGCTCAACGCCTACTTTCACCAGCCTGAGGC
CATCGAGGAGTTCCCTGTGCCAGCCCTGCACCACCCAGTGTTCCAGCAGGAGAGCTTT
ACCCGCCAGGTGCTGTGGAAACTGCTGAAAGTGGTGAAGTTCGGAGAGGTCATCAGCT
ACAGCCACCTGGCCGCCCTGGCCGGCAATCCCGCCGCCACCGCCGCCGTGAAAACCG
CCCTGAGCGGAAATCCCGTGCCCATTCTGATCCCCTGCCACCGGGTGGTGCAGGGCG
ACCTGGACGTGGGGGGCTACGAGGGCGGGCTCGCCGTGAAAGAGTGGCTGCTGGCC
CACGAGGGCCACAGACTGGGCAAGCCTGGGCTGGGTTAGcgcg**G**gtttcg**A**tggcggggcgggg
cctgggaacggatgagaccccgccttccgtaggccaccgtctctccagaggccacgcccctgcacctcagtggccttggctggt
gaggccctgcctcctgcccctggaaaagccatagagggtccggtgccctgggcatgggctgggcaggcctgcctgggaggcc
gggtcgtagcctctggtgtctgtgcaaaatacagtgtgggatgggctctcaaaagcacacgcgccctcggcgcacacctggggct
gagggcgcctctctccgcggaccccgccctcagcagcgggcccattggcagctggcgacaccctgctcgtgccacacacggtg
ggctgagctccgacgaaaggctcagcccccatcgctgctaaatacccgatttagaatctccctgattcttccccgagggccccac
aggccaggcgcccctccccccaattgaaccggttgccaaacaagtttctgtccttgcccttccttctgggactttagtcggaaccg
gcctagtcaagaagggggggggtgtcccagagccaagggcccccatcccatcttttctccaaatccagtcaggaaataagaga
tcagaaactaggtaggagggagaaagcgagaatatgaacgtgcatttctgagtgtcctgtgcttgtgataaagggatgacccttttt
ctaattggctttgaaccacagcatgtcccctgaatgtcacgtgcagtgacagggtagaattcaggctcttacataggatccaagtttc
atccatggacagggagttcagaggctacattccatacattgaggggggacttgatcccgaccacactggagctcaggagggagt
gatttacactttagggcaacaaacatggctaaaggggaagtggtc-3'

Location PCR and Genotyping

For genotyping, genomic DNA (gDNA) was isolated from tail biopsies using a genomic DNA isolation kit (Nexttec, #10.924).

For the diagnostic location PCR, 20 µL reactions were prepared using 1 µL clean gDNA (15-80ng), 4 µL PrimerSet (4 pmol final each), 4 µL 5X Reaction Buffer (Finnzymes #F-524), 0.4 µL PhireHot-Start II Taq DNA Polymerase (Finnzymes #F-122L), 1 µL 10 mM dNTPs (Bioline #DM-515107), 4 µL Hi-Spec Additive (Bioline #HS-014101) and 5.6 µL H2O. Thermocycler parameters: 98°C for 5 min, (98 °C for 45 s, 64 °C for 30 s, 72 °C for 60 s) repeated for 34 cycles, final step at 72 °C for 10 min.

For routine genotyping, 20 µL PCR reactions were prepared using 1 µL clean gDNA (15-80ng), 4 µL PrimerSet (4 pmol final each), 4 µL 5X Reaction Buffer (Biozym #331620XL), 0.2 µL Hot-Start Taq DNA Polymerase (Biozym #331620XL), 1 µL 50 mM MgCl2 (AGCTLab stock) and 9.8 µL H2O. Thermocycler parameters: 96°C for 3 min, (94 °C for 30 s, 62 °C for 60 s, 72 °C for 60 s) repeated for 32 cycles, final step at 72 °C for 7 min.

Diagnostic, location PCR

Location1 = 1506bp

5'-AGAAGATGGGCGAGAGGATC-3'
sense_Upstream_HDR1-Munc13-1 (Location 1)

5'-CCCGCCATCGAAACCCGCGCTAACCCAGCCCAGGCTTG-3'
asense-Munc13-1-3’-UTR-SNAP-tag (Location 1)

Location2 = 1564bp

5'-GCGGCGGAAGCGGCGGTAGCATGGACAAAGACTGCGA-3'
sense-Linker-SNAP-tag (Location 2)

5'-ATCTTGGCTCTGTCAGTCAC-3'
asense_Downstream_HDR1-Munc13-1 (Location 2)

Genotyping strategy

Primer sequences:

WT band = 162bp

5'-AGCGCAGCAACGATGAGGTG-3'
senseExon44_Munc13-1

5'-GAGAGACGGTGGCCTACGGA-3'
3‘-UTR_Munc13-1

SNAP-tag KI = 225bp

5'-AGCGCAGCAACGATGAGGTG-3'
senseExon44_Munc13-1

5'-CTTTGCCCAGGAAGATGATACGG-3'
asense_SNAP-tag

General Chemistry

All chemical reagents and anhydrous solvents for synthesis were purchased from commercial suppliers (Sigma-Aldrich, Fluka, Acros, Fluorochem, TCI) and were used without further purification.

NMR spectra were recorded in deuterated solvents on a Bruker AVANCE III 600 equipped with a CryoProbe calibrated to residual solvent peaks (^{1}H in ppm): MeOD-d_4 (3.31). Multiplicities are abbreviated as follows: s = singlet, d = doublet, t = triplet, q = quartet, p = pentet, h = heptet, br = broad, m = multiplet. Coupling constants *J* are reported in Hz. Spectra are reported based on appearance, not on theoretical multiplicities derived from structural information.

UPLC-UV/Vis for purity assessment was performed on an Agilent 1260 Infinity II LC System equipped with Agilent SB-C18 column (1.8 µm, 2.1 × 50 mm). Buffer A: 0.1% FA in H_2O Buffer B: 0.1% FA acetonitrile. The typical gradient was from 10% B for 0.5 min -> gradient to 95% B over 5 min -> 95% B for 0.5 min -> gradient to 99% B over 1 min with 0.8 mL/min flow. Chromatograms were imported into GraphPad Prism 10 and plotted.

High resolution ESI-MS spectra were recorded on a Waters H-class instrument equipped with a quaternary solvent manager, a Waters sample manager-FTN, a Waters PDA detector and a Waters column manager with an Acquity UPLC protein BEH C18 column (1.7 µm, 2.1 mm x 50 mm). Samples were eluted with a flow rate of 0.3 mL/min. The following gradient was used: A: 0.01% FA in H_2O; B: 0.01% FA in MeCN. 5% B: 0-1 min; 5 to 95% B: 1-7min; 95% B: 7 to 8.5 min. Mass analysis was conducted with a Waters XEVO G2-XS QTof analyzer.

SBG-SiR-d12 analysis

^{1}H NMR spectrum:

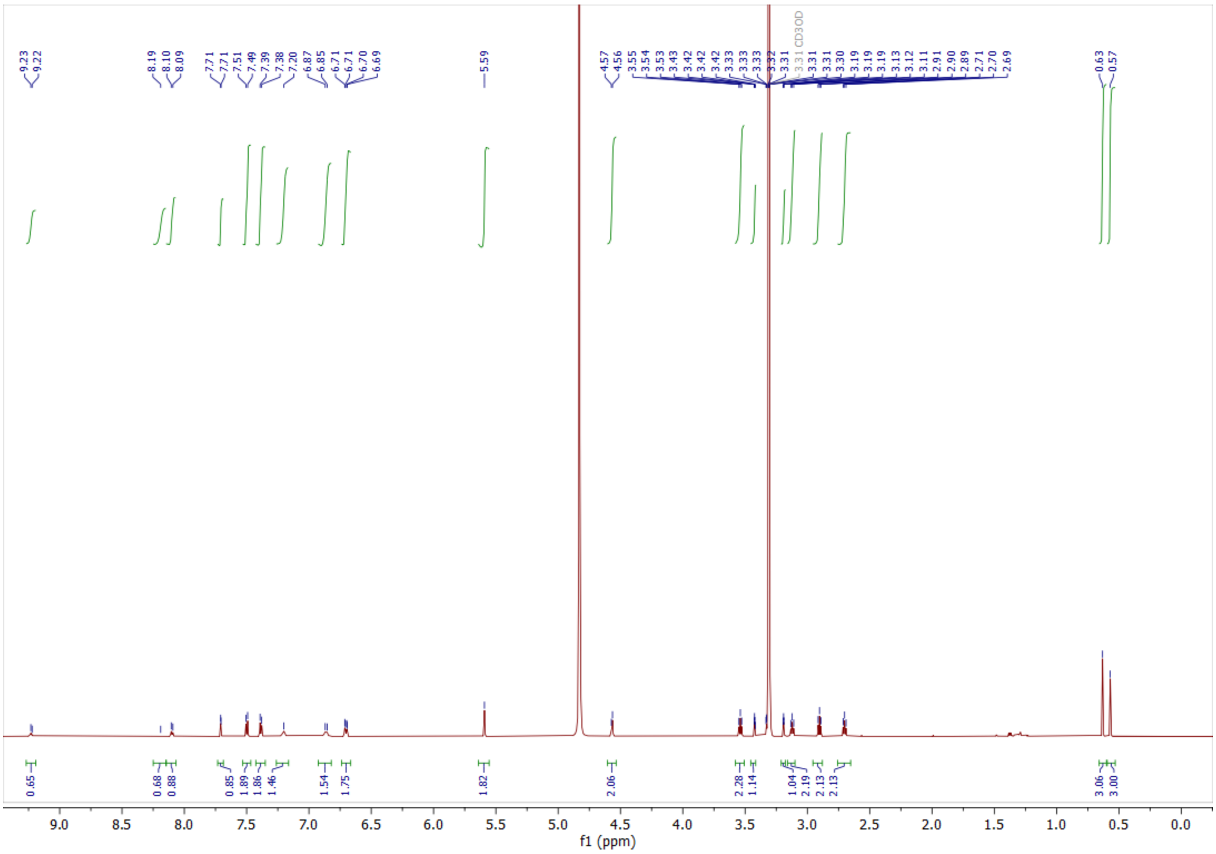

LCMS trace:

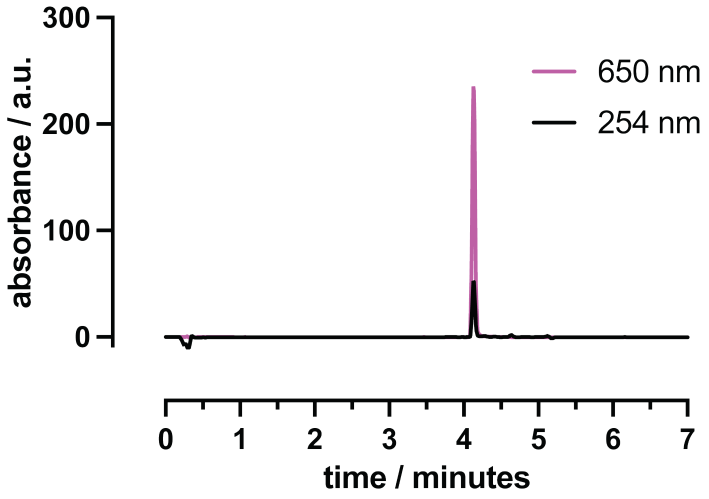

Excitation and emission spectra:

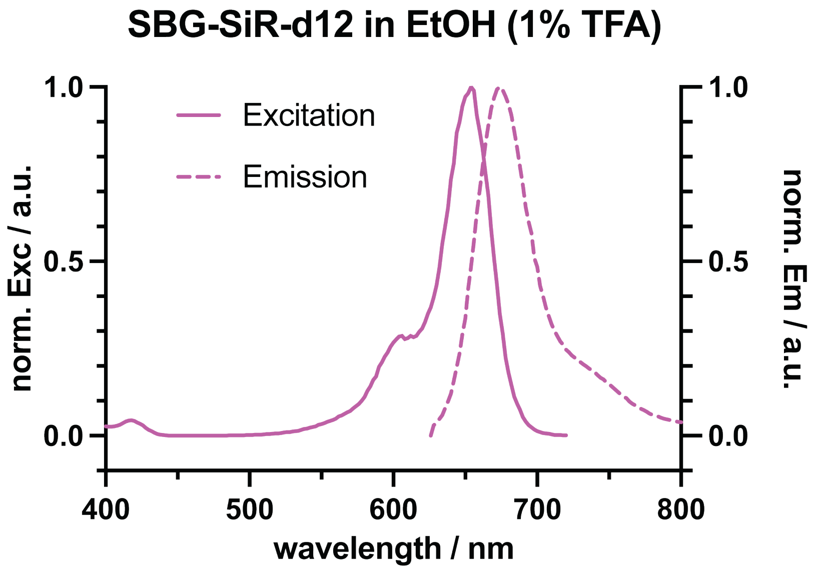

Emission and excitation spectra of SBG-SiR-d12 were determined at a concentration of 100 nM in EtOH + 1% TFA using an Infinite 200 PRO (Tecan) and plotted in GraphPad Prism 10.
